# Supplementary figures and images for: Effect of Moringa Oleifera fortified porridge consumption on protein and vitamin A status of children with cerebral palsy in Nairobi, Kenya: A randomized controlled trial
Source: PLOS Glob Public Health. 2022 Nov 4;2(11):e0001206. doi: 10.1371/journal.pgph.0001206 (PMC10021702; doi:10.1371/journal.pgph.0001206)

**S1 Fig: Pictures of intervention product**


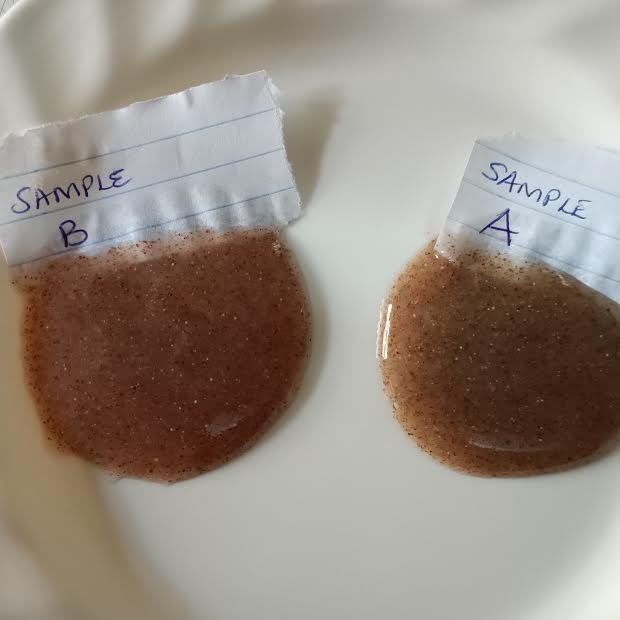


**Key**

A-Fortified Porridge B- Unfortified Porridge

Supplement: S1 Fig — (DOCX) [file pgph.0001206.s002.docx]
