# Supplementary material for: Effect of Moringa Oleifera fortified porridge consumption on protein and vitamin A status of children with cerebral palsy in Nairobi, Kenya: A randomized controlled trial
Source: PLOS Glob Public Health. 2022 Nov 4;2(11):e0001206. doi: 10.1371/journal.pgph.0001206 (PMC10021702; doi:10.1371/journal.pgph.0001206)
Supplement: S1 Table — (DOCX) [file pgph.0001206.s003.docx]

**S1 Table: Socio-demographic characteristics of caregivers**

|  | Intervention,  N=57 | Control,  N=56 | Total  N=113 | p-value |
| --- | --- | --- | --- | --- |
| Caregiver characteristics |  |  |  |  |
| Age mean (SD) | 35.8(6.5) | 37.8(6.5) |  | 0.103^a^ |
| Marital status  Married  Single  divorced  cohabiting  total | **n (%)**  26(45.6)  14(24.6)  6(10.5)  11(19.3)  57(100.0) | **n (%)**  21(37.5)  16(28.6)  12(21.4)  7(12.5)  56(100) | **n (%)**  47(41.6)  30(26.5)  18(15.9)  18(15.9)  113(100) | 0.315^b^ |
| Education  No formal  primary  secondary  college  university  total | 2(3.5)  21(36.8)  14(24.6)  17(29.8)  3(5.3)  57(100) | 1(1.8)  24(42.9)  19(33.9)  9(16.1)  3(5.4)  56(100) | 3(2.7)  45(39.8)  33(29.2)  26(23.0)  6(5.3)  113(100) | 0.442^b^ |
| Occupation  Salaried employment  Self-employment  other  total | 36(63.2)  10(17.5)  11(19.3)  57(100) | 31(55.4)  16(28.6)  9(16.1)  56(100) | 67(59.3)  26(23.0)  20(17.7)  113(100) | 0.377^b^ |
| No. of Children  1  2  3  4  total | 5(8.8)  22(38.6)  15(26.3)  15(26.3)  57(100) | 3(5.4)  16(28.6)  21(37.5)  16(28.6)  56(100) | 8(7.1)  38(33.6)  36(31.9)  31(27.4)  113(100) | 0.478^b^ |
| Household characteristics |  |  |  |  |
| Gender of Household Head  Male  female  Total | 37(64.9)  20(35.1)  57(100) | 29(51.8)  27(48.2)  56(100) | 66(58.4)  47(41.6)  113(100) | 0.184^b^ |
| Wealth quintile  Poorest  Poor  Middle  Least poor | 13(22.8)  12(21.1)  24(42.1)  8(14.0) | 15(26.8)  12(21.4)  25(44.6)  4(7.1) |  | 0.685^b^ |
| Mean Wealth Index score | 0.12 | -0.12 |  | 0.203^a^ |
| Child characteristics |  |  |  |  |
| Gender Boys n (%)  Girls n (%) | 31 (54.4)  26 (45.6) | 28 (50)  28 (50) |  | 0.708^b^ |
| Mean age +SD  Median (Range) | 106.98±25.8  120 (62- 141) | 108.89±22.5  121 (61-140) |  | 0.676^a^  0.778^c^ |

- ^a^t-test
- ^b^Chi-square test
- ^c^Median test
- SD = Standard Deviation
