# Supplementary material for: Effect of Moringa Oleifera fortified porridge consumption on protein and vitamin A status of children with cerebral palsy in Nairobi, Kenya: A randomized controlled trial
Source: PLOS Glob Public Health. 2022 Nov 4;2(11):e0001206. doi: 10.1371/journal.pgph.0001206 (PMC10021702; doi:10.1371/journal.pgph.0001206)
Supplement: S2 Table — (DOCX) [file pgph.0001206.s004.docx]

# S2 Table: Pilot study results

| **Variable** | **description** |  | **Experimental** | **control** | **p-value** |
| --- | --- | --- | --- | --- | --- |
| **Caregiver characteristics** | | | | | |
| **Mean age** | Age (years) |  | 35(4) | 38.5(3.3) | 0.131 |
| **Marital status** | married | Baseline | 3(42.9) | 3(42.9) | 0.282 |
|  | single | Baseline | 1(14.3) | 1(14.3) |  |
|  | divorced | Baseline | 1(14.3) | 3(42.9) |  |
|  | cohabiting | Baseline | 2(28.6) | 0(0.0) |  |
|  | total | Baseline | 7(100) | 7(100) |  |
| **Education** | No formal | Baseline | 0(0.0) | 1(14.3) | 0.248 |
|  | primary | Baseline | 6(85.7) | 3(42.9) |  |
|  | secondary | Baseline | 1(14.3) | 2(28.6) |  |
|  | college | Baseline | 0(0.0) | 1(14.3) |  |
|  | university | Baseline | 0 | 0 |  |
|  | total | Baseline | 7(100) | 7(100) |  |
| **Occupation** | Salaried employment | Baseline | 3(42.9) | 1(14.3) | 0.452 |
|  | Self-employment | Baseline | 2(28.6) | 4(57.1) |  |
|  | Other | Baseline | 2(28.6) | 2(28.6) |  |
| **Household characteristics** | | | | | |
| **HH Gender** | Male | Baseline | 5 | 3 | 0.589 |
|  | Female | Baseline | 2 | 4 |  |
| **Mean WI score** |  | Baseline | -0.47(0.25) | -0.32(0.27) | 0.305 |
| **Wealth quintiles** | poorest |  | 3(42.9) | 2(28.6) | 0.486 |
|  | poor |  | 2(28.6) | 2(28.6) |  |
|  | middle |  | 1(14.3) | 3(42.9) |  |
|  | Least poor |  | - | - |  |
|  |  |  |  |  |  |
| **Child characteristics** | | | | | |
| **Age (months)** | Mean age | Baseline | 113.3(18.2) | 109.3(23.8) | 0.485 |
|  |  | End line | 116.3(18.2) | 112.3(23.8) | 0.485 |
|  | Median age | Baseline | 121.0(84-135) | 118.0(61-133) | 0.567 |
|  |  | End line | 124.0(87-138) | 121.0(64-136) | 0.567 |
| **Nutrient intakes** | Energy | Baseline | 1245.92(159.0) | 1283.72(594.8) | 0.626 |
|  |  | End line | 1684.46(254.2) | 1209.11(356.3) | 0.02 |
|  | Energy fulfil | Baseline | 61.19(12.4) | 63.05(17.5) | 0.621 |
|  |  | End line | 82.73(8.0) | 59.38(29.1) | 0.020 |
|  | Proteins | Baseline | 33.44(17.6) | 31.82(5.6) | 0.409 |
|  |  | End line | 46.80(5.7) | 34.21(7.2) | 0.006 |
|  | Protein fulfil | Baseline | 55.64(11.9) | 52.94(9.1) | 0.417 |
|  |  | End line | 77.87(29.4) | 56.92(9.2) | 0.006 |
|  | Vitamin A | Baseline | 413.06(107.3) | 415.38(361.1) | 0.535 |
|  |  | End line | 645.46(237.5) | 419.24(127.7) | 0.041 |
|  | Vitamin A fulfil | Baseline | 49.17 (6.1) | 49.45(15.1) | 0.593 |
|  |  | End line | 76.84(22.8) | 49.90(17.6) | 0.042 |
| **Nutritional status** | Serum albumin(g/dL) | Baseline | 3.31(1.53) | 3.10(1.44) | 0.493 |
|  |  | End line | 4.43(2.56) | 3.23(1.81) | 0.038 |
|  | Serum retinol (µmol/L) | Baseline | 0.39(0.16) | 0.37(0.17) | 0.463 |
|  |  | End line | 0.725(0.12) | 0.41(0.17) | 0.032 |
|  | Mean BMI (boys) | Baseline | 15.29(1.5) | 15.25(1.7) | 0.733 |
|  |  | End line | 20.00(6.4) | 15.86 (5.7) | 0.016 |
|  | Mean BMI (girls) | Baseline | 16.34 (6.3) | 16.50(5.8) | 0.856 |
|  |  | End line | 22.80(2.6) | 16.48(1.7) | 0.022 |
|  | Mean BMI Z-scores (boys) | Baseline | -1.1 SD | -1.3 SD | 0.234 |
|  |  | End line | ±1.2 SD | -1.1 SD | 0.049 |
|  | Mean BMI Z-scores (girls) | Baseline | -0.16 | .-0.25 SD | 0.311 |
|  |  | End line | ±2.3 SD | ±0.24 SD | 0.047 |
| **Morbidity** | Children who fell ill in previous 2 weeks | Baseline | 7(100) | 6(85.7) | 0.265 |
|  |  | End line | 0(0) | 5(71.4) | 0.044 |

**NB:**

- Values in parenthesis are percentages
