# Supplementary material for: Effect of Moringa Oleifera fortified porridge consumption on protein and vitamin A status of children with cerebral palsy in Nairobi, Kenya: A randomized controlled trial
Source: PLOS Glob Public Health. 2022 Nov 4;2(11):e0001206. doi: 10.1371/journal.pgph.0001206 (PMC10021702; doi:10.1371/journal.pgph.0001206)
Supplement: S1 File — (DOC) [file pgph.0001206.s005.doc]

**EFFECT OF *MORINGA OLEIFERA* CONSUMPTION ON PROTEIN AND VITAMIN A STATUS OF CHILDREN WITH CEREBRAL PALSY IN NAIROBI KENYA: A RANDOMIZED CONTROLLED TRIAL**

**MALLA JANET KAJUJU (MSc. FND)**

**H87/38307/2016**

**DEPARTMENT OF FOOD, NUTRITION AND DIETETICS**

**A RESEARCH PROPOSAL SUBMITTED IN FULFILMENT OF THE REQUIREMENTS FOR THE AWARD OF THE DEGREE OF DOCTOR OF PHILOSOPHY IN FOOD, NUTRITION AND DIETETICS, IN THE SCHOOL OF APPLIED HUMAN SCIENCES OF KENYATTA UNIVERSITY**

**JULY 2017**

# DECLARATION

This proposal is my original work and has not been presented for a degree in any other University.

Signature: -------------------------------- Date: ------------------

Malla Janet Kajuju:

Admission Number: H87/38307/2016

Supervisors: This proposal has been submitted for review with our approval as

University supervisors:

Signature: -------------------------------- Date: ------------------

Sophie Ochola (PhD),

Department of Food, Nutrition and Dietetics,

Kenyatta University

Signature: ----------------------------------Date: -----------------

Irene Ogada (PhD),

Department of Food, Nutrition and Dietetics,

Kenyatta University

Signature: ------------------------------------ Date: ------------------

Ann Munyaka (PhD),

Department of Food, Nutrition and Dietetics,

Kenyatta University

# TABLE OF CONTENTS

[DECLARATION ii](#__RefHeading___Toc483218216)

[TABLE OF CONTENTS iii](#__RefHeading___Toc483218217)

[LIST OF TABLES v](#__RefHeading___Toc483218218)

[LIST OF FIGURES vi](#__RefHeading___Toc483218219)

[ABBREVIATIONS AND ACRONYMS vii](#__RefHeading___Toc483218220)

[OPERATIONAL DEFINITION OF TERMS viii](#__RefHeading___Toc483218221)

[ABSTRACT ix](#__RefHeading___Toc483218222)

[CHAPTER ONE: INTRODUCTION 1](#__RefHeading___Toc483218223)

[1.1 Background to the study 1](#__RefHeading___Toc483218224)

[1.2 Statement of the problem 3](#__RefHeading___Toc483218225)

[1.3 Purpose of the study 4](#__RefHeading___Toc483218226)

[1.4 Objectives of the study 5](#__RefHeading___Toc483218227)

[1.5 Hypotheses 5](#__RefHeading___Toc483218228)

[1.6 Significance of the study 6](#__RefHeading___Toc483218229)

[1.7 Delimitations of the study 6](#__RefHeading___Toc483218230)

[1.8 Limitations of the study 6](#__RefHeading___Toc483218231)

[1.9 Assumptions of the study 7](#__RefHeading___Toc483218232)

[1.10 Conceptual framework 7](#__RefHeading___Toc483218233)

[CHAPTER TWO: LITERATURE REVIEW 8](#__RefHeading___Toc483218234)

[2.1 Prevalence of cerebral palsy 8](#__RefHeading___Toc483218235)

[2.2 Nutritional requirements of children with cerebral palsy 8](#__RefHeading___Toc483218236)

[2.3 Complications of cerebral palsy and nutritional effects on the children 10](#__RefHeading___Toc483218237)

[2.4 Nutrient composition of finger millet (Eleusine coracana) 10](#__RefHeading___Toc483218238)

[2.5 Nutrient composition of Moringa oleifera leaves 11](#__RefHeading___Toc483218239)

[2.6 Interventions with Moringa oleifeira leaf powder 12](#__RefHeading___Toc483218240)

[2.7 Summary of literature review 13](#__RefHeading___Toc483218241)

[CHAPTER THREE: METHODOLOGY 14](#__RefHeading___Toc483218242)

[3.1 Study design 14](#__RefHeading___Toc483218243)

[3.1.1 Phase one (product development) 14](#__RefHeading___Toc483218244)

[3.2 Nutrient and anti-nutrient analysis of flours and porridges 16](#__RefHeading___Toc483218245)

[3.3 Phase two of the study (intervention phase) 16](#__RefHeading___Toc483218246)

[3.4 Study area 17](#__RefHeading___Toc483218247)

[3.5 Target population 18](#__RefHeading___Toc483218248)

[3.5.1 Inclusion criteria 18](#__RefHeading___Toc483218249)

[3.5.2 Exclusion criteria 18](#__RefHeading___Toc483218250)

[3.6 Sample size 19](#__RefHeading___Toc483218251)

[3.7 Randomization and recruitment 19](#__RefHeading___Toc483218252)

[3.8 Blinding 20](#__RefHeading___Toc483218253)

[3.9 Description of the interventions 20](#__RefHeading___Toc483218254)

[3.10 Research instruments 21](#__RefHeading___Toc483218255)

[3.10.1 Questionnaire 21](#__RefHeading___Toc483218256)

[3.10.2 Baseline questionnaire 22](#__RefHeading___Toc483218257)

[3.10.3 Other questionnaires 22](#__RefHeading___Toc483218258)

[3.10.4 Equipment 23](#__RefHeading___Toc483218259)

[3.11 Pilot study 23](#__RefHeading___Toc483218260)

[3.12 Validity and reliability of research instruments 24](#__RefHeading___Toc483218261)

[3.12.1 Validity 24](#__RefHeading___Toc483218262)

[3.12.2 Reliability 24](#__RefHeading___Toc483218263)

[3.13 Selection and training of the research team 24](#__RefHeading___Toc483218264)

[3.13.1 Selection criteria for research assistants 24](#__RefHeading___Toc483218265)

[3.13.2 The research team 25](#__RefHeading___Toc483218266)

[3.13.3 Training of research team 25](#__RefHeading___Toc483218267)

[3.14 Data collection procedures 26](#__RefHeading___Toc483218268)

[3.14.1 Dietary intake 26](#__RefHeading___Toc483218269)

[3.14.2 Weight measurements for the children 26](#__RefHeading___Toc483218270)

[3.14.3 Mid-upper arm circumference (MUAC) measurement 27](#__RefHeading___Toc483218271)

[3.14.4 Blood sample collection 27](#__RefHeading___Toc483218272)

[3.15 Biochemical analysis 28](#__RefHeading___Toc483218273)

[3.16 Data analysis 28](#__RefHeading___Toc483218274)

[3.17 Logistics and ethical considerations 29](#__RefHeading___Toc483218275)

[REFERENCES 31](#__RefHeading___Toc483218276)

[Appendix B1: Preparation of M. oilefera leaf powder 44](#__RefHeading___Toc483218277)

[Appendix B2: Fermentation of finger millet 44](#__RefHeading___Toc483218278)

[Appendix C: Proximate analysis 45](#__RefHeading___Toc483218279)

[Appendix D: Nutrient profile form 51](#__RefHeading___Toc483218280)

[Appendix E: Acceptability of the M. oleifera fortified millet porridge by the caregiver’s of children 52](#__RefHeading___Toc483218281)

[Appendix F1: Laboratory request form 53](#__RefHeading___Toc483218282)

[Appendix F2: Biochemical determination of retinol 54](#__RefHeading___Toc483218283)

[Appendix F3: Biochemical determination of serum albumin 54](#__RefHeading___Toc483218284)

[Appendix G: Questionnaires 56](#__RefHeading___Toc483218285)

[Appendix H: Morbidity 65](#__RefHeading___Toc483218286)

[Appendix I: Research budget 66](#__RefHeading___Toc483218287)

[Appendix J: Work plan 67](#__RefHeading___Toc483218288)

# LIST OF TABLES

Table 2.1 Nutrient Requirements (RDAs) for children aged 5-11years…………………..9

Table 2.2 Nutritional value of finger millet per 100g of edible portion…………….…...11

Table 2.3 Nutrient comparison in 100g *M. oleifera* leaf powder...…………..……….....12

Table 2.4 Percentage RDA of different nutrients provided in 25g of dried *M. oleifera*

leaf powder for children……………………………………………………...12

Table 3.1 Study variables………………………………………………………..……….17

# LIST OF FIGURES

Figure 1.1: Factors affecting protein and vitamin A status. Adapted from Kerac et al.

(2014)………….…………..……………..……………………………………………………………………………7

Figure 3.1: A schematic representation of the study design, intervention group and

Follow- up………………………………………………......………………21

# ABBREVIATIONS AND ACRONYMS

**AGADA:** Alternative Action for African Development

**CP:** Cerebral Palsy.

**CWS:** Church World Service

**ECD:** Early Childhood Development

**FAO:** Food Agricultural Organization

**IMAM:** Integrated Management of Acute Malnutrition

**MUAC:** Middle Upper Arm Circumference

**OMD:** Oral Motor Dysfunction

**PWDs:** Persons with Disabilities

**SDG:** Sustainable Development Goals

**UNICEF:** United Nations Children’s Fund

**VAD:** Vitamin A Deficiency

**WHO:** World Health Organization

# OPERATIONAL DEFINITION OF TERMS

**Caregiver**: A person directly in charge of feeding and general nursing care of the child with Cerebral Palsy

**Cerebral Palsy:** A condition that affects body movements and muscle co-ordination

**Control group:** Study participants who will receive fermented millet flour porridge only from the research.

**Dietary patterns:** Refers to types of foods eaten regularly by children with CP.

**Experimental group:** Study participants who will receive dried *moringa oleifera* leaves/

millet blend porridge.

***Moringa Oleifera:*** A plant species from the *Moringaceae* family that will be used in the study

**Physical disability:** Limitation of the physical activity of limbs and fine bones

**Protein Status:** In this study it will be determined by serum albumin level.

**Participants:** Children with Cerebral Palsy and their Caregivers.

**Socio-economic status:** Individual’s status as determined by education, occupation and income level

**Study group:** Children with Cerebral Palsy (5-11 Years) involved in the study

**Vitamin A Status:** Determined by serum levels for retinol in the blood

# ABSTRACT

Cerebral palsy (CP) is a physical disability affecting 0.4% children globally. Children with CP experience feeding problems like swallowing and chewing. Many are unable to request for food leading to poor intake of nutrients, resulting in malnutrition. Malnutrition in CP is largely related to inadequate protein intake and is consequently associated with vitamin A deficiency. A diet that is nutrient-dense, yet easy to chew and swallow is therefore essential to improve the protein and vitamin A status of these children. Research has shown *Moringa oleifera* leaf powder to be effective in alleviating protein and vitamin A deficiencies. The purpose of the study is to test the effect of *M. oleifera* consumption on the protein and vitamin A status of children with CP. This study will be conducted at Little Rock Day Care Centre located in Kibera Informal Settlement, Nairobi. This will be a randomized controlled trial with a sample of 114 children with CP randomly placed into 2 study groups (intervention and control). Participants in the control group will be fed on fermented finger millet porridge while those in the intervention group will receive fermented finger millet porridge fortified with dried *M. oleifera* leaf powder daily for 3 months*.* Data collection (qualitative and quantitative) will be conducted by interviewing caregivers on socio-economic and demographic characteristics and dietary intake of children with CP. Anthropometric measurements of children (weight and MUAC) will be taken. The Statistical Package for Social Sciences (SPSS) will be used for data analysis. Descriptive statistics such as: means, frequencies and standard deviations will be used to describe characteristics of the study population on; age, sex, education level, socio-economic status, medical history. Nutri-survey software will be used to analyze dietary nutrient intake and results compared with the recommended daily allowances by UNICEF/WHO (2002). Weight-for-age indices will be used to express the nutritional status of the children. WHO Child Growth Standards (2006) will be used to interpret the nutritional status of the children. Levels of serum albumin and retinol will be determined through biochemical tests. Nutrient and anti-nutrient profile of finger millet flour and *M. oleifera* leaf powder will be determined through proximate analysis. Chi-square tests will be used to determine the association between consumption of *M. oleifera* fortified porridge and levels of serum albumin and retinol. Tukey’s studentized range test will be used to measure statistical difference in colour, texture, taste, and acceptability of the different formulations of porridges. Independent t-test will be conducted to test for significant differences between the intervention and control group for continuous variables such as levels of serum albumin and retinol therefore testing the effectiveness of the intervention. The significance level will be p<0.05. Qualitative data will be categorized into themes and patterns then coded. The findings of this study will contribute to the on-going research efforts on food-based approaches in preventing and managing malnutrition among children, especially those with disabilities like CP, using locally available indigenous foods.

# CHAPTER ONE: INTRODUCTION

## 1.1 Background to the study

Cerebral palsy (CP) is a disorder which causes limitation in movement of limbs due to damage in the central nervous, or dysfunction originating early in life (Elkamil et al., 2011). It is a physical disability that affects 0.4% of children globally (Yeargin-Allsop et al., 2008). According to the World Disability Report (2011), 15% of the global population constitutes persons with disability. In Kenya, the overall disability rate is 4.6% translating to 1.7 million Persons with disabilities. Of this, the largest proportion is physical impairment, amounting to 24% (413,698), of which CP is inclusive (KNBS; NCAPD, 2007).

In developed countries, CP affects between 0.12% and 0.30% of children (Hustad et al., 2011). In the U.S. prevalence is estimated at 0.24% (Hirtz et al., 2007). In Sweden it is 0.22% (Himmelmann et al., 2010) and in Australia it is between 0.20% and 0.25% (Reddihough & Collins, 2003). In the developing countries there is limited data regarding the prevalence of CP. A study conducted in South Africa indicated high prevalence rates between 1.0 and 8.0 % (Christianson et al., 2002). Studies conducted in Kenya by Auka and Afedo in 1986 and Kennedy in 1990, showed that CP affected about 0.25% of children. There is limited data on the current prevalence rate of CP in Kenya.

According to Henderson (2007), 40-60% of children with CP are malnourished. Stallings et al., (1996) studied 154 children with CP between 2-17 years of age from Philadelphia and found that 67% were malnourished. Ifeyinwa et al., (2010), studied 98 children with CP from Nigeria and found that 36% were malnourished. A study conducted by Koriata (2012), in Kenya among children with CP attending Kenyatta National Hospital outpatient clinic, showed that 70.3% were malnourished. Feeding dysfunction is common in children with CP resulting to poor health and nutritional status (Fung et. al., 2002). Because of these challenges in feeding, children with CP need interventions that will enhance their nutritional status. Alternative foods that are nutrient-dense and affordable could therefore help in alleviating malnutrition. One of such food that could serve as a valuable source of nutrient is *Moringa oleifera* leaf powder*.* Research has shown *M. oleifera* leaf powder to be effective in reducing nutritional deficiencies such as protein and vitamin A deficiency (Fuglie, 2001; Price, 2002).

*Moringa oleifera,* (drumstick tree), belongs to the *Moringaceae* family. It grows fast in the drought areas of the tropics in Africa, South America and India (Lim, 2012). It is economically grown in most parts of Kenya (Schmidt & Mwaura, 2010). The *Moringaceae* family consists of 13 species and *M. oleifera* is the most widely known species. All parts of the tree are edible. According to Fahey (2005) there is substantial health benefit in consumption of *M. oleifera* in cases of starvation. Because of its high protein and micronutrient content in the leaves, *M. oleifera* is reported to prevent malnutrition (Anjorin et al., 2010). According to Fuglie (2001), 100g of the fresh *M. oleifera* leaves contain 6.7g of protein, 440 mg of Calcium, 6.8 mg of vitamin A and 220mg of vitamin C. Organoleptic properties tested on mother panelists have shown high preference in taste, colour and accceptability of fortified *M. oleifera* finger millet porridge compared with traditional finger millet porridge (Barugahara et.al., 2015).

## 1.2 Statement of the problem

According to Hung et al., (2003), a large number of children with special needs are malnourished. Malnutrition in CP is related to inadequate protein intake mostly due to impaired chewing and swallowing (Stallings et al., 1996). Protein foods like meat are hard to chew and swallow; a typical diet for these children mostly consists of foods that are easily mashed like bananas, potatoes and beans to enhance swallowing. Plant proteins are harder to digest and have low utilizable protein value compared to meat (Young and Pellett, 1994). Adequate protein is also necessary for the mobilization of vitamin A from the liver to the blood stream and therefore protein malnutrition will result in vitamin A deficiency (Ikekpeazu et al., 2010). These factors therefore inform the focus on protein and vitamin A status of children with CP in this study.

According to Fung et al., (2002), feeding difficulties interferes with the child’s ability to adequately consume essential nutrients required for growth therefore resulting to malnutrition. According to Munk (1994), 30 to 80% of children with CP often have feeding difficulties and most are unable to request for food and drink, therefore lacking adequate nutrition which negatively affects their growth. Children aged between 5-11 years old are vulnerable because of their rapid growth rate. They need more attention and care of physical and mental development which are directly related to nutritional status (Nandy et al., 2005).

The draft Kenya National disability policy report (2011), and the Disability Act (2003) provides for the rights and privileges of PWDs such as education, health and other rehabilitation services, and funding for PWDs in general. However, both the Act and policy do not enlist CP as one of the physical disabilities that is known to exist nor include nutrition intervention as one of the services to be provided to PWDs. For effective rehabilitation, especially of children with CP, nutrition intervention component should be included among others.

Children with CP also have a right to improved nutrition and healthy lives as enshrined in the Children’s Act (2010) and Sustainable Development Goals (SDG)-2 and SDG-3. The Children’s Act (2010) CAP 141 states that a disabled child shall have the right to be accorded appropriate medical treatment, special care, education and training free of charge whenever possible. SDG-2, seeks to “end hunger, achieve food security and improved nutrition”, while SDG-3 seeks to “ensure healthy lives and promote well-being for all at all ages”. Although very few studies have been conducted in Kenya on nutrition status of CP, (Koriata, 2012) there is no tangible intervention study known to the researcher that has been conducted to improve the nutritional status of children with CP in Nairobi County.

According to Zongo et al., (2013), giving *M. oleifera* leaf powder in where there is deficiency of nutrients improves nutritional status of the children. Studies addressing nutritional appropriateness of local foods to treat malnutrition in children specifically those with CP are limited. The lack of rigorous and systematic tests of the nutritional efficacy of local food supplements to treat malnutrition therefore constitutes a gap this study proposes to address.

## 1.3 Purpose of the study

The purpose of the study is to determine the effect of *M. oleifera* consumption on protein and vitamin A status of children with CP in Nairobi County.

## 1.4 Objectives of the study

The specific objectives will be to:

1. Develop porridge from dried *M. oleifera* leaves mixed with fermented Finger millet (*Eleusine coracana*) flour.
2. Establish the nutrient and anti-nutrient profile of dried *M. oleifera* leaves, fermented finger millet flour and the composite porridge made from dried *M. oleifera* leaves and fermented millet flour.
3. Determine the acceptability of the porridge developed from dried *M. oleifera* leaves mixed with fermented finger millet flour.
4. Determine the effect of consumption of *M. oleifera* fortified millet porridge on levels of serum albumin and retinol in children with CP 5-11 years of age in Nairobi County.

## 1.5 Hypotheses

This study will test the following hypotheses;

Ho_1_: There is no significant difference in the nutrient profiles of the porridge made from fermented finger millet flour and the composite porridge made from fermented finger millet flour fortified with dried *M. oleifera* leaves powder.

Ho_2_: There is no significant difference in the acceptability of the porridge developed from dried *M. oleifera* leaves mixed with fermented finger millet flour and fermented finger millet flour.

Ho_3_: Consumption of *M. oleifera* fortified millet porridge does not significantly improve level of serum albumin and retinol in children aged 5-11years with CP in Nairobi County.

1.6 Significance of the study

The study is consistent with the objectives of national and global initiatives and strategies related to Integrated Management of Acute Malnutrition. The study findings will therefore be beneficial to the Ministry of Health, the Association for People with Special Needs and other stakeholders concerned with the health of children. The study will contribute to the on-going research efforts on food-based approaches in preventing and managing malnutrition especially among children with disabilities using locally available indigenous foods. It will also contribute valuable information that may contribute towards achieving the goal of having “Improved livelihoods for the vulnerable persons, such as the physically disabled, both at household, community and national levels” as enshrined in the Kenya Vision 2030.

## 1.7 Delimitations of the study

Children with Cerebral Palsy aged 5-11 years in Nairobi County will constitute the focus of study therefore the findings can only be generalized to areas and to children with similar characteristics

## 1.8 Limitations of the study

All children with CP aged 5-11 years regardless of the form and severity of CP will be included into the study but it is assumed that all these children will be able to consume the porridge provided in the study. The study will be limited because the children participating are exposed to other foods at home and therefore not within the control of the study. However, dietary intake will be used to monitor and record intakes of food consumed at home.

## 1.9 Assumptions of the study

The participants will accept and feed on the developed food product and that major changes in the intake of food will be minimal among the participants during the period of study.

## 1.10 Conceptual framework

The conceptual framework (Figure 1.1) has been adapted from Kerac et al., (2014). It explains the relationship between dependent (levels for serum albumin and retinol, morbidity, weight and wasting) and the independent variable (consumption of fermented finger millet porridge fortified with *M. oleifera* leaf powder).

| Morbidity prevalence- type and duration of illness |
| --- |

| -Dietary intake  -Socio- demographic &economic  characteristics |
| --- |

| Consumption of fermented finger millet porridge fortified with *M. oleifera* leaf powder |
| --- |

| - levels for serum albumin and retinol  -Weight gain  -Wasting |
| --- |

Figure 1.1: Factors affecting protein and vitamin A status.

Adapted from Kerac et al., (2014)

The primary outcome variables in this study are protein and vitamin A status as determined by levels for serum albumin and retinol respectively, whereas the secondary outcome variables are weight gain and wasting. These are influenced by the consumption of fermented finger millet porridge fortified with *M. oleifera* leaf powder, dietary intake, morbidity prevalence, feeding difficulties, and socio-demographic and economic factors.

# CHAPTER TWO: LITERATURE REVIEW

## 2.1 Prevalence of cerebral palsy

Cerebral palsy (CP) refers to a disorder which causes limitation in movement of limbs due to a central nervous lesion, damage or dysfunction originating early in life (Elkamil et al., 2011). It is a physical disability that affects 0.4% of children globally (Yeargin-Allsop et al., 2008). In developed countries, international assessments show that CP affects between 0.12% and 0.30% children (Hustad et al., 2011). Studies conducted in Kenya by Auka and Afedo in 1986 and Kennedy in 1990, showed that CP affected about 0.25% of children. In the same study, CP was noted to be the second most common neurological condition causing physical impairments after poliomyelitis in Kenya (Auka and Afedo, 1985; Kennedy, 2001). Current data on prevalence of CP in Kenya is lacking.

## 2.2 Nutritional requirements of children with cerebral palsy

Research conducted on nutritional requirements of children with CP is limited, therefore nutritional requirements for children with no disabilities is applicable to children with CP who have no limitations in movement (Fung et al., 2002). A healthy child requires 2000 kcal/day (Table 2.1). However, energy requirements of children with severe CP who utilize a wheelchair for mobility have been reported to be between 60 and 70% lower than healthy typically developing children (Walker et al., 2012) , so their energy requirements is between 1200-1400 kcal/day. Table 2.1 shows the requirements (RDAs) for some nutrients for children without disabilities aged 5-11years.

Table 2.1 Requirements (RDAs) for some nutrients for children aged 5-11years.

| Nutrient | RDA |
| --- | --- |
| Energy | 2000kcal/day |
| Protein | 1g/kg /day |
| Vitamin A | 700μg RE |
| Vitamin C | 45mg |
| Iron | 10mg |
| Zinc | 10mg |

Source: Fung et al., (2002)

**2.2.1 Protein requirements**

Adequate protein intake is required to build and repair tissue, for adequate growth and development in childhood. There is currently no data available for protein requirements of children with CP therefore their requirements do not differ from the typically normal developing children and recommendations can be applied (1.0 g/kg of bwt/day) as shown in Table 2.1. Severely malnourished children with CP, require additional protein for ‘catch up’ growth. Overall, an intake of 2.0 g/ kg of bwt/day of protein intake is considered to be sufficient in these instances (Pencharz, 2010).

**2.2.2 Vitamin A requirements**

Vitamin A is in form of β - carotene in plant foods and is a precursor of vitamin A (retinol) in the body. β - carotene is the most common form of carotenoids found in vegetables and the most common type of pro-vitamin A. Vitamin A is essential for proper functioning of immune system. According to GoK (2008) adequate vitamin A intake has been found to reduce child mortality and morbidity significantly. Deficiency of vitamin A occurs if serum retinol is < 0.7 μmol/L for a long period. It leads to a compromised immune system thus increased vulnerability to infections (UNICEF, 2003). According to Ngare et al., (2000), Vitamin A utilization can be impaired by frequent infections and under nutrition. Globally about 500 million children become blind every year of which 230 million are in the developing world (WHO, 2009). For children 5-11years the RDA is 700μg RE /day (Samson-Fang et al., 2002) as shown in Table 2.1.

## 2.3 Complications of cerebral palsy and nutritional effects on the children

Children with CP often have feeding difficulties influencing their nutrient intake and hence their growth (Fung et al., 2002). Available literature suggests that all children with CP are at risk of malnutrition despite the degree of motor impairment,. Stallings et al., (1996) studied 154 children with CP between 2-17 years of age from Philadelphia and found that 67% were malnourished. Ifeyinwa et al., (2010), studied 98 children with CP from Nigeria and found that 36% were malnourished. A study conducted by Koriata (2012), in Kenya among children with CP attending Kenyatta National Hospital outpatient clinic, showed that 70.3% were malnourished. National statistics on levels of malnutrition among this population is limited.

## 2.4 Nutrient composition of finger millet (Eleusine coracana)

Finger millet, *E. coracana* belongs to the family of poaceae gramineae. It is also known as *wimbi, mugimbi* (Kenya).It is mainly consumed in India and Africa. Finger millet is a good source of nutrients and in general they are rich sources of vitamin B but available data are very meager on vitamin content of millets (Mbithi et al., 2000). Finger millet is a popular complementary food but is limited in essential amino acids (Singh and Raghuvanshi, 2012). Nutritive value of millet is also limited by high concentrations of anti-nutrients such as phytates and tannins, and these reduce bioavailability of some nutrients especially iron and zinc (Bachar et al., 2013).Table 2.2 shows the nutritional value of finger millet per 100g of fresh edible portion.

Table 2.2 Nutritional value of finger millet per 100g of fresh weight edible portion

| Nutrient | Amount |
| --- | --- |
| Protein | 7.6g |
| Fat | 1.5g |
| Carbohydrate | 88g |
| Calcium | 370mg |
| Vitamins- A (carotenoids) | 0.48mg |
| Thiamine (B1) | 0.33mg |
| Ribofavin (B2) | 0.11mg |
| Niacin: (B3) | 1.2mg |
| Fiber | 3g |

Source: Gopalan et al. (2009)

## 2.5 Nutrient composition of Moringa oleifera leaves

*Moringa oleifera* (drumstick tree) is an edible plant and the most widely cultivated species of the genus *Moringa*, which is the only genus in the family *Moringaceae*. English common names include: *moringa,* drumstick tree, horseradish tree, ben oil tree, or benzoil tree. *M. oleifera* is used as human food and for medicinal purposes worldwide (Fuglie, 2001). *M. oleifera* can be used as a source of nutrients (Appendix F4). The leaves are the most nutritious part of the plant, being a significant source of B vitamins, vitamin C, provitamin A (carotenoids) e.g. beta-carotene, vitamin K, manganese, and [protein](http://en.wikipedia.org/wiki/Protein), among other essential nutrients (Carlquist, & Olson 2001; Peter, 2008). Table 2.3 below shows the Nutrient composition in 100g of *M. oleifera* leaves compared to common foods.

*Table 2.3 Nutrient comparison in 100g M. oleifera*

| Nutrient | Common Foods(mg)/100g fresh weight | Fresh *Moringa* Leaves | Dried *Moringa* Leaves |
| --- | --- | --- | --- |
| Vitamin A (mg) | Carrots 1.8 | 6.8 | 18.9 |
| Calcium (mg) | Milk 120 | 440 | 2003 |
| Potassium (mg) | Bananas 88 | 259 | 1324 |
| Protein (g) | Yoghurt 3.1 | 6.7 | 27.1 |
| Vitamin C (mg) | Orange 30 | 220 | 17.3 |

Source: Fuglie (2001)

Malnourished children should consume 25g of *M. oleifera* leaf powder each day (Fuglie, 2001). According to FAO/WHO standards, these amounts provide the following RDAs for the nutrients shown in Table 2.4.

Table 2.4 Percentage RDA of different nutrients provided in 25g of dried *M. oleifera* leaf powder for children

| Nutrient | %RDA in 25g powder for children |
| --- | --- |
| Protein | 42 |
| Calcium | 125 |
| Magnesium | 61 |
| Potassium | 41 |
| Iron | 71 |
| Vitamin A | 310 |
| Vitamin C | 22 |

Source: (Fuglie, 2001).

## 2.6 Interventions with Moringa oleifeira leaf powder

*Moringa* trees have been used to alleviate malnutrition, especially among infants and nursing mothers (Fuglie, 1999). According to Fahey (2005), consumption of *M. oleifera* leaf powder during starvation has substantial health benefits. *M. oleifera* is reported to prevent malnutrition because of the high protein and micronutrient content of the leaves (Anjorin et al., 2010). Research conducted by Zongo et al., (2013), in Burkina Faso showed that malnourished children fed on *Moringa* leaf powder as a nutritional supplement recorded a higher average daily weight gain ( 8.9 ± 4.30g/kg/day) compared to (5.7 ± 2.72 g/kg/day) in children not receiving the supplement.

Other intervention programmes in Africa have been implemented to promote the use of *M. oleifera* as a traditional leafy vegetable to improve nutritional status. The government of Malawi encouraged the use *M. oleifera* as a potential solution to Vitamin A deficiency (Babu, 2000). A study conducted in Niger to determine the nutrient content of leaves of seven wild plants showed that, *M. oleifera* leaves contained the highest overall protein quantity in comparison with the WHO protein standard (Freiberger *et al.*, 1998).

## 2.7 Summary of literature review

Malnutrition in CP is related to inadequate nutrient intake some of them being protein and Vitamin A. Feeding difficulties are very common in children with CP contributing to 48% suffering from growth retardation. Focus on protein and Vitamin A in this study is therefore informed by fact that children with CP are malnourished hence are usually deficient in protein and Vitamin A. The literature reviewed shows that giving *M. oleifera* leaf powder, as a nutritional supplement improves the nutritional status of the individuals. The intervention studies using *M. oleifera* have mainly targeted children with no disabilities. There is need therefore to assess the effect of *M. oleifera* in combating malnutrition in children with disabilities and specifically those with CP, which this study intends to address.

# CHAPTER THREE: METHODOLOGY

## Study design

The study will comprise of two phases. Phase one of the study will comprise the development of porridge made from fermented millet flour fortified with dried *M. oleifera* leaves powder. The porridge will be assessed for its nutrient and anti-nutrient content and acceptability among children with CP. In phase two of the study, a randomized controlled trial will be carried out to determine the effect of consumption of the fortified porridge on protein and vitamin A status of children with CP.

## Phase one (product development)

- - 1. **Preparation of *Moringa oilefera* leaf powder**

In this study, the *Moringa* species used will be *M. oleifera* obtained from Kibwezi, Makueni County*.* The method of preparation will be solar drying which is described in Appendix B1.

- - 1. **Preparation and fermentation of millet flour**

In this study, the millet species used will be Finger Millet (*Eleusine coracana*)*.* Millet used in this study will be fermented before milling as described in Appendix B2.

- - 1. **Formulation of fermented finger millet and *M. oleifera* leaf powder porridge**

Fermented finger millet will be fortified with *M. oleifera* leaf powder after milling. Pearson square method (Wagner & Stanton, 2009) will be used to determine the proportion for mixing. The product formulation will be aimed at providing adequate protein which will be the target primary nutrient.

- - 1. **Preparation of the porridges**

Porridge preparation will be done at the rehabilitation centre. This study will aim at providing one cup (250 mls) of porridge once daily to children with CP (5-11yrs). The mixed flour samples will be weighed into a saucepan. Water at room temperature will be added and mixed with the flour to make slurry in the ratio of 100g flour to 120ml water. Twenty five grammes (25 g) sugar will be added the slurry to improve the taste and 10g oil to enhance vitamin A absorption. The slurry will then be added into 750ml of boiling water, stirred and left to boil for ten minutes. This will make 1litre of porridge and the quantities will be multiplied by 13 to produce 13 litres for each group (control and intervention).The porridges will be left to cool at room temperature.

- - 1. **Acceptability of porridges**

Acceptability and sensory evaluation of the fermented millet flour and dried *M. oleifera* leaf/ fermented finger millet porridge will be carried out using 5 point hedonic scale (1 = extremely dislike, 2= moderately dislike, 3= neither dislike, 4= moderately like and 5= extremely like). Organoleptic qualities such as colour, texture, taste and overall acceptability will be evaluated by an untrained sensory panel comprising of 6 (10%) randomly selected caregivers as recommended by Abebe et al., (2006). This is because the children with CP might not provide a reliable response. A sensory evaluation form (Appendix E) will be provided to the panelists. Clear explanations on the study objectives and procedure for sensory evaluation will be given to caregivers.

## Nutrient and anti-nutrient analysis of flours and porridges

The dried *M. oleifera* leaf powder, fermented finger millet flour, *M. oleifera* -finger millet blend and fermented finger millet porridge will be analyzed for selected macronutrients and micronutrients. (Appendix D)

- - 1. **Proximate analysis**

Proximate analysis will be used to determine the moisture, ash, fat, protein, fibre. The standard method of Association of Official Analytical Chemists, (AOAC, 2005) will be applied. Carbohydrate content will be obtained by difference method as described by FAO (2010), Shahnawaz et al. (2009) and James (1995). Procedures are described in (Appendix C).

## Phase two of the study (intervention phase)

**3.4.1 Design of the intervention study**

This will be a randomized controlled trial to test the effect of consumption of *M. oleifera* fortified finger millet porridge on protein and vitamin A status in children with CP. Randomization aims at ensuring the participants on different treatments are comparable with respect to baseline characteristics, as well as known and unknown risk factors (Machin et al.,2007). There will be two study arms, control group (receiving fermented finger millet porridge) and intervention group (receiving fermented finger millet porridge fortified with *M. oleifera* leaf powder). Data collection, analysis and presentation will use quantitative and qualitative techniques.

**3.4.2 Study variables**

The primary outcomes in this study will be; protein and vitamin A status, whereas the secondary outcomes will be weight gain and wasting. The indicators for the primary outcomes will be; levels for serum albumin and serum retinol, consumption of fermented finger millet porridge fortified with *M. oleifera* leaf powder, morbidity prevalence, as well as socio-demographic and economic characteristics among caregivers, dietary intake as shown in Table 3.1

| **Outcome variables** | **Measurement indicators for outcome variables** | **Independent variables** | **Indicators for independent variables** |
| --- | --- | --- | --- |
| **Primary outcomes**:   - Vitamin A status - Protein status | - Serum retinol level - Serum albumin | - Consumption of fermented finger millet porridge with and without  *M. oleifera* leaf powder |  |
| **Secondary outcomes:**  Weight gain  Wasting | - Monthly weight gain - Based on MUAC | - Dietary intake - Socio-demographic and economic characteristics of caregivers | Frequency of meals,  Types and amounts foods eaten  Age, sex of the participant, Education level, occupation and income of the parents |
|  |  | - Morbidity prevalence | Frequency and type of illness Based on a two week recall |

Table 3.1 Study variables

## Study area

The study will be conducted at Little Rock Day Care Centre located in Kibera informal settlement in Nairobi County. Kibera comprises of 13 villages, located approximately 5km southwest of the Nairobi city centre. The Little Rock day care centre provides early childhood education for children 1-13 years of age and has over 791 children. Among these are 180 children with special needs (CP, hearing impairment and Down syndrome). Data on prevalence of children with CP in Kibera is limited. The Day Care Centre will be purposively selected because it gives nutrition support in terms of providing finger millet porridge at ten o’clock and lunch to all the children enrolled in the centre. It also caters for children with special needs in terms of providing other rehabilitation services e.g. occupational therapy and early childhood education. These children mainly come from Kibera.

## Target population

The target population will be children 5-11 years of age with cerebral palsy attending Little Rock Day Care Centre in Kibera informal settlement, Nairobi County. In addition, the director and staff at the Centre will also be targeted.

### Inclusion criteria

Children 5-11 years of age attending Little Rock Day Care Centre and have been diagnosed by a medical doctor to have CP.

### Exclusion criteria

Children with CP with chronic ailments such as; cardiovascular diseases, cancer, renal dysfunction and liver problems diagnosed by a medical doctor will be excluded since these would independently affect food intake. Additionally, those whose caregivers decline to participate or give consent will be excluded from the study.

## Sample size

This is calculated as shown below, for a two sided significance of 5%, power of 80% and a moderate anticipated standardized effect of 0.05 as recommended by Cohen, (1988) and Machin et al., (2007). An increment of 10% to the sample size will be done to cater for attrition.

n =2[(*a+b*)^2^ σ^2^]

(μ_1-_ μ_2_)^2^

n = the sample size in each of the groups

μ_1_ = population mean in treatment Group 1

μ_2_ = population mean in treatment Group 2

μ_1_ − μ_2_ = the difference the investigator wishes to detect =11

σ^2^ = population variance =20

a = conventional multiplier for alpha = 0.05

b = conventional multiplier for power = 0.80

2× [(1.96 + 0.842)^2^ × 20^2^] / 11^2^ = 51.9, round off to 52

This means that a sample size of **52** participants per group is needed to answer the research question.This sample size will be inflated by 10% of 52 (5) and therefore the final sample size will be; 52+5=**57** participants per group totaling to =**114**

## Randomization and recruitment

Randomization will be conducted by an independent biostastician using a formula generated in Microsoft office Excel 2008. Participants will be randomized on a ratio of 1:1 into the control and intervention group respectively and given random numbers by the researcher (Figure.3.1).

## Blinding

This will be a double blind study in order to control for bias. The biostatistician in charge of conducting randomization of the study sample population, the participants, research assistants, cooks, and laboratory technician will be blinded of the study treatment and hypotheses. It is only the researcher who will be aware of the treatment and the groups to enable accuracy during the intervention.

## Description of the interventions

- - 1. **The control group**

The participants in this group will be fed on the standard finger millet porridge usually fed to the children at the centre. This will be prepared and administered at the centre by the researcher assisted by the research assistants, caterer and cooks at the centre and given in a cup equivalent to 250ml at 10 am once daily, five days a week, for a period of three months.

- - 1. **Intervention group**

Participants in this group will be fed on the *M. oleifera* fortified millet porridge prepared at the centre and administered by the researcher assisted by the research assistants, caterer, and cooks. It will be given in a cup equivalent to 250 ml at ten o’clock in the morning once daily, five days a week, for three months. There will be no sharing of cups or porridge. Left-over porridge will be discarded to avoid cross contamination.

| RECRUITMENT OF STUDY PARTICIPANTS   - Informed consent and recruitment of study participants at Little Rock ECD centre - Eligibility criteria:   Children with CP who are not diagnosed with HIV/AIDS; not on multivitamin supplements; heart diseases; renal dysfunction, hepatitis, whose caregiver has given consent |
| --- |

| Control group | Intervention group |
| --- | --- |

| Baseline data collection on;  Socio-demographic and economic characteristics, medical history, weight and MUAC measurement, dietary and morbidity patterns, serum levels for protein, retinol and β – carotene, |
| --- |

| INTERVENTION PHASE |
| --- |

| Control group  -No intervention from the research team; fermented finger millet porridge will be given in a 250ml cup once daily, five days a week for three months | Intervention group  -Fermented *M. oleifera* fortified finger millet porridge will be given in a 250ml cup once daily, five days a week for three months |
| --- | --- |

| DETERMINATION OF NUTRITIONAL OUTCOMES |
| --- |

| Control group  - Data collection on dietary and morbidity, weight and MUAC (6 weeks and then at 12 weeks) -Serum levels for albumin and retinol will be determined at the end of the intervention. | Intervention group  - Data collection on dietary and morbidity, weight and MUAC (6 weeks and then at 12 weeks) - Serum levels for albumin and retinol will be determined at the end of the intervention. |
| --- | --- |

**Figure 3.1:** A schematic representation of the study activities

## Research instruments

### Questionnaire

There will be several questionnaires as follows:

### Baseline questionnaire

This will be a researcher-administered questionnaire (Appendix G1&2) and will be administered to caregivers of both study groups upon recruitment. The questionnaire will consist of closed and open ended questions that will be used to solicit information on; demographics, socio-economic characteristics, medical history, dietary and morbidity patterns and similarly, information will be sought on body measurements (anthropometry) where weight and MUAC will be measured and 7 day food frequencies.

### Other questionnaires

These will be administered to all participants at the end of 6 weeks and then at 12 weeks in the study. The questionnaires will consist of closed and open ended questions that will solicit information on dietary intake and morbidity prevalence. Information will be also be sought on body measurements (anthropometry) where weight and MUAC will be measured (Appendix G3-I, G3-II, G4).

#### 3.10.3.1 Food frequency questionnaire

This will be a 7 day Food Frequency administered to the caregivers to solicit information on the frequency and types of food items consumed by the children (Appendix G3-I).

#### 3.10.3.2 24-hour recall

This will be administered to the caregivers to solicit information on the frequency of meals, types and amounts foods consumed by the children (Appendix G3-II).

#### 3.10.3.3 Key informant interview guide

The Key Informant Interview Guide (KII) will consist of questions that the researcher will use while conducting interviews. The guide will solicit information from the director of the centre and her assistants on the; management, number of staff and qualifications, financial support, child population, attendance, feeding, benefits and services provided at the centre (Appendix G5).

### 3.10.4 Equipment

#### 3.10.4.1 Anthropometric equipment

Salter scale (Model 2006) and standard electronic scale (Seca model 770; Seca Hamburg, Germany) will be used to take weight while MUAC tapes used to take middle upper arm circumference measurements.

#### 3.10.4.2 Blood collection equipment

The following equipment will be used ; vacutainer needles , tubes, vacutainer holder, tourniquet, disinfection swabs, micropore tape, adhesive dressing, rubber gloves, disposable syringes, pillow or other support, separate stoppers for opened vacuum tubes and non-vacuum tubes and needle disposal box. transfer and storage tubes (freezable), disposable pipettes or pipettes with changeable apex, centrifuge with swinging bucket rotor, timer, racks for tubes, aluminium foil, set of labels with identification codes (freezable), refrigerator and a freezer. Safe disposal of needles and transportation of blood samples to the laboratory will be done to ensure that there is no cross-contamination.

## Pilot study

A pilot study will be conducted on seven children with CP outside the study area at Challenge Programme Centre situated in Shauri, Moyo, Nairobi County. The participants in this centre have the same characteristics as the study sample and will not be included in the main study. The importance of piloting is to evaluate the study procedures and data collection tools. Data collection tools will be tested for clarity, consistency, coherence and sensitivity. Piloting will be conducted for a period of 1 month. Data collection be reviewed based on the feedback, study procedures adjusted accordingly and data will be analysed and a report written.

## Validity and reliability of research instruments

### Validity

The study supervisors will evaluate the questionnaires and give feedback to the researcher. Also, standard forms for evaluating organoleptic qualities and recording of the 24-hour recall will be used, and these have already been validated.

### Reliability

The test-retest technique will be employed. Administration of each questionnaire will be done twice to the same participant at an interval of two days. During piloting, reliability of the instruments will be tested using Cronbach alpha coefficient and data computed after the pilot study. Reliabilities ranging from 0.7 onwards are considered acceptable (Fraenkel and Wallen, 2000). During the training and pilot study, the research assistants will be provided with practical sessions on data collection procedures which will be standardized to reduce on errors.

## Selection and training of the research team

### Selection criteria for research assistants

Nutritionists should be holders of Bsc. or Diploma (Food, Nutrition and Dietetics), fluent in both English and Kiswahili languages. They should have work experience of at least 3 months in a nutrition related field and also willing to work in the study for 3 months. Experience in conducting surveys will be an added advantage. Requirements for a Phlebotomist, laboratory technician, food technologist will be aBsc. or Diploma and for a cook, caterer/cateress will be a certificate or diploma in their related fields of study.

### The research team

This team will consist of the principle researcher, who will train the team, oversee data collection and intervention, four nutritionists; 2 nutritionists will assist the researcher in administering the baseline and monthly questionnaires, 2 nutritionists will involved in the preparation and service of fermented finger millet porridge fortified with  *M. oleifera* leaf powder at the centre. A cook to prepare the porridge and a caterer/cateress to serve it to the children, a phlebotomist to draw blood from the participants for biochemical analysis, a laboratory technician to treat and analyze the blood samples drawn from the participants, and a food technologist to conduct nutrient analysis of *M. oleifera* leaf powder and the flour formulations.

### Training of research team

The team with the exception of the phlebotomist, food technologist and laboratory technician will be trained by the researcher. The training will be conducted at the centre for a period of 3 days through lecturers, demonstrations and role play. The content will be on; objectives of the study, recruitment of the study participants, inclusion and exclusion criteria, taking of anthropometric measurements, administering the questionnaires and research ethics. The training will be conducted at the centre for a period of 3 days through lecturers, demonstrations and role play.

## Data collection procedures

Baseline data will be collected immediately after recruitment into the study (Appendix G1&G2). A questionnaire will be administered to the caregiver by the researcher to provide information on the child’s dietary patterns based on the 7-day food frequency, 24-hour recall (Appendix G3-I &II). The director of the centre and her assistants will also be interviewed using Key Informant Interview Guide (Appendix G5) to provide general information about the centre, child- enrollment, attendance and any difficulties encountered when feeding the CP children.

### 3.14.1 Dietary intake

Dietary intake for children with CP will be assessed using a 24-hour recall and food frequency (Appendix G3-I). The 24-hour recall will be conducted for two days in a week and the average calculated to determine the amount and type of foods taken per day and the adequacy of the nutrients. The quantity of food consumed will be estimated using the commonly used household equipment. A seven (7) day Food Frequency Questionnaire (Appendix G3-II) will be used to interview caregivers on household food intake. This procedure will be conducted at baseline, 6 weeks and then at 12 weeks to monitor and assess any changes.

### 3.14.2 Weight measurements for the children

This will be measured to the nearest 0·1 kg on standard electronic scales. A bathroom weighing scale (Salter) will be used. Participants unable to stand due to impairment will be weighed together with the caregiver and then the caregiver’s weight will be deducted from the value of the combined weight. The difference derived will be the weight of the child. The measurements will be taken twice and, if the variance between the two measurements is within acceptable levels, an average will be determined to ensure accuracy. This exercise will be conducted at baseline and at the end of the intervention (Appendix G4).

### 3.14.3 Mid-upper arm circumference (MUAC) measurement

This will be taken on the left arm which is considered to be less active and therefore has less muscle. The child will be in a seated or lying position and relaxed. The arm mid-point will be established by measuring the distance between tip of the shoulder and the elbow using the MUAC tape. The circumference will be measured using the same MUAC tape. The arm of the child will be straightened, the tape wrapped around the arm at the midpoint with the numbers right side up and the tape flat around the skin with correct tension. Read the measurement to the nearest 0.1 cm. The measurements will be taken twice and, if the variance between the two measurements is within acceptable levels, an average determined to ensure accuracy. This exercise will be conducted at baseline and at the end of the intervention (Appendix G4).

### 3.14.4 Blood sample collection

Blood samples will be drawn by a certified phlebotomist under the standard operational procedures. Five millilitres of blood will be collected through venipuncture of a peripheral vein using disposable syringes. Blood will be collected mostly in the mornings. The specimens will be collected in trace-element free tubes and immediately wrapped in aluminium foil, to shield from light and stored at 4 °C until centrifugation and then put in portable freezers for transportation to the laboratory for biochemical analysis. This procedure will be done at baseline and at the end of the intervention.

## Biochemical analysis

Biochemical analysis will be done to determine levels for serum albumin and retinol in the study participants. Sample extraction steps will be performed in glass apparatus covered with aluminium foil to minimize exposure to light. Extracts for serum albumin and retinol will be completed on the same day and injected into the HPLC column to reduce exposure time of the sample. Procedure for analysis is described in appendix F2&F3

## Data analysis

Data will be entered into MS Access 2008 database and exported to Excel 2008. A spread sheet will then be generated to counter-check each entry against the questionnaire after which the data will be exported to Statistical Package for Social Sciences (SPSS) version 19.0 for analysis. Descriptive statistics such as: means, frequencies and standard deviations will be used to describe the characteristics of the study population on; age, sex, education level, socio-economic status and medical history of the children. Dietary nutrient intake will be analyzed using Nutri-survey software and compared with the recommended daily allowances as recommended by, UNICEF/WHO (2002). Weight-for-age indices will be used to express the nutritional status of the children. WHO Child Growth Standards (2006) will be used to interpret the nutritional status of the children because this is what is currently used in Kenya and globally. Levels for serum albumin and retinol will be determined through biochemical tests. Nutrient and anti-nutrient profile of finger millet flour and *M.oleifera* leaf powder will be determined through proximate analysis.

Inferential statistics will be used to determine relationships and associations between consumption of *M. oleifera* and serum levels for albumin and retinol. Chi-square tests will be used to determine relationships between categorical variables like sex and morbidity. Tukey’s studentized range test will be used to measure statistical difference in colour, texture, taste, and acceptability of the different formulations of porridges. Independent t-test will be conducted to test for significant differences between the intervention and the control groups for continuous variables such as age and weight, serum levels of albumin and retinol and hence test the effectiveness of the intervention. Pearson product moment correlation (r) will be used to determine the relationship between non-categorical variables like dietary intake and serum levels and morbidity prevalence. The level of significance will be set at p<0.05. Qualitative data in terms of words or phrases will be categorized into themes and patterns, then coded and conclusions made.

## Logistics and ethical considerations

Authority to conduct the research will be sought from Kenyatta University Graduate School and the ethical clearance will also be sought from Kenyatta University Ethical and Review Committee (KUERC). A research permit will then be obtained from National Commission of Science, Technology and Innovation (NACOSTI). Authority to conduct the study will be sought from the management of Little Rock ECD Centre and also of Nairobi County authorities.

The children with CP will be recruited into the study upon the informed consent of their parents/guardians. Participation will be voluntary. The parents/guardians will be free to withdraw their children at any point in time when they choose to. They will also be informed that their unwillingness to have their children participate in the study will not in any way affect the services the children receive at the Little Rock Day Care centre. The staff from the centre will also be recruited upon their informed consent. The information obtained from participants will be treated as confidential and will only be used for the purpose of the study. The participants will be identified by unique numbers and not by names. All the filled in questionnaires will be kept under lock and key and only the principal researcher will have access to the cupboard. The findings will be used only for the purpose of the study. At the end of the study, participants in the control group will be provided with *M. oleifera* fortified millet porridge for three months equivalent period of the intervention time for ethical reasons. Children found malnourished will be referred to a health facility for treatment.

# REFERENCES

Abebe Y., Stoecker B., Hinds J., and Gates G. (2006). Nutritive Value and Sensory Acceptability of Corn- and Kocho-Based Foods Supplemented With Legumes for Infant Feeding in Southern Ethiopia. AJFAND. Vol 1, No 1, 2006.

Anjorin T.S., Ikokoh P, Okolo S. (2010).Mineral composition of Moringa oleifera leaves pods and seeds from two regions in Abuja, Nigeria. Int. J. Agric. Biol.; 12:431-434.

Anwar F, Latif S, Ashraf M, Gilani A.H. (2007). Moringa oleifera: A Food Plant with Multiple Medicinal Uses. Phytotherapy Research. 21:17-25.

AOAC. (1990). Official methods of analysis of the Association of Official Analytical Chemists. *Association of Official Analytical Chemists, Arlington, VA, USA*.

AOAC. (2000). Official methods of analysis of the Association of Official Analytical Chemists. Retrieved 16 March 2015, from <http://scholar.google.com/scholar>

AOAC. (2005). *Official and tentative methods of analysis* (Vol. 1). Association off. Agri. Chemists.

ASPEN Board of Directors and the Clinical Guidelines Task Force (2002). Guidelines for the use of Parenteral and enteral nutrition in adult and pediatric patients. J Parenter Enteral Nutr 26 (1 Suppl), 1SA-138SA. (Erratum in J Parenter Enteral Nut 2002; 26:144).

Auka, M.N., & Afedo, N.E (1985). On the job in servicing and seminar programme for the teachers of physically handicapped. Nairobi: Ministry of Education, Science &Technology

Babu S. (2000).Rural nutrition interventions with indigenous plant foods-a case study vitamin A Deficiency in Malawi. Biotechnology, Agronomy, Society, and Environment. 4(3):169-179.

Bachar K, Mansour E, Ben Khaled A, Mabrouka A, Haddad M, Ben-Yahya L, El-Jarray N, Ferchichi A (2013). Fiber content and mineral composition of the finger millet of the Oasis of Gabes Tunisia. J. Agric. Sci. 5 (2): 219

Bhatt A, Singh V, Shrotria P.K, Baskheti DC (2003). Coarse Grains of Uttaranchal: Ensuring sustainable Food and Nutritional Security. Indian Farmer’s Digest. pp. 34-38.

Brooks J, Day S, Shavelle R, Strauss D. (2011).Low weight, morbidity, and mortality in children with Cerebral palsy: new clinical growth charts. Pediatrics; 128: e299-e307.

Barugahara Evyline Isingoma, Mbugua Samuel, Karuri Edward ,Gakenia Maina(2015).

Improving the nutritional value of traditional finger millet porridges for children aged 7-24 months in Bujenje County of Western Uganda. African Journal of Food Science, Vol. 9(8), pp. 426-436

Campanozzi A, Capano G, Miele E. (2007). Impact of malnutrition on gastrointestinal disorders and gross motor abilities in children with cerebral palsy. Brain Dev.; 29:25- 29 Canada, CA. 910pp.

Center for Disease Control and Prevention, (2012). Cerebral Palsy, [Online]. USA: Center for Disease Control and Prevention.

Cerebral Palsy Association Eastern Cape (2013)Hambisela programme making waves in the Eastern Cape http://www.discovery.co.za/discovery

Chandra R.K, Kumari S. (1994). Nutrition and immunity: an overview. J Nutr.; 124:1433S- 1435S

Christianson, A. Zwane, M. Manga, P. Rosen, E. Venter, A. Downs, D. Kromberg, J.( 2002). Children with intellectual disability in rural South Africa: prevalence and associated disability. Journal of Intellectual Disability Research 46(Pt 2) Feb: 179-186

Cohen (1988). Statistical power analysis for the behavioural sciences (2nd edition). New Jersey: Lawrence Earbaun

Cronbach, L. J., & Shavelson, R. J. (2004). My current thoughts on coefficient alpha and successor procedures. *Educational and Psychological Measurement*, *64*(3), 391–418.

Day S, Strauss D, Vachon P, et al. (2007).Growth patterns in a population of children and adolescents with cerebral palsy. Dev Med Child Neurol.; 49:167-171

Duker J.S, Yoshida Y, Niki E, Rasmussen H, et al., (2009). Oxidative stress and antioxidant status in older adults with early cataract. Eye (Lond) 23: 1464-1468.

Elkamil A, Andersen,G.L, Hagglunad G, Lamvik T, Skranes J, and Vik, T, (2011). Prevalence of hip dislocation among children with cerebral palsy in regions with and without a surveillance programme: a cross sectional study in Sweden and Norway. BioMedCentral Musculoskeletal disorder, 12:284.

Fahey J. (2005). Moringa oleifera: A Review of the medical Evidence for Its Nutritional Therapeutic and Prophylactic Properties. Part 1. Trees for Life Journal.

Faria A.V, Hoon, A, Stachinko, Miller E. J (2011). Quantitative Analysis of Brain Pathology Based on MRI and Brain Atlases - Applications for Cerebral Palsy. Neuroimage, 54(3):1854-1861.

FAO. (2010). Methods of Food, Analysis. Agriculture and Consumer Protection. [www.fao.org/docrep/006N5022e03.htm Accessed on 7/112009](http://www.fao.org/docrep/006N5022e03.htm Accessed on 7/112009).

FAO. (2010): Food and Agriculture Organization of the United Nations. Retrieved from http://faostat. fao.org/site/5 67/defult.aspxJ /ancor.

FAO (1991). Amino Acid Scoring Pattern. In: Protein quality evaluation, FAO/WHO Food and Nutrition Paper, Italy. pp. 12-24.

Fraenkel J and Wallen N (2000). How to Design and Evaluate Research in Education*.* (4^th^ Edition)*.* New York: McGraw Hill.

Freiberger C.E (1998). "Nutrient content of the edible leaves of seven wild plants from Niger." Plant Foods for Human Nutrition: 57-69.

Fuglie L.J (1999). The Miracle Tree: Moringa oleifera: Natural Nutrition for the Tropics. Church World Service; Dakar, Senegal.

Fuglie L (2001). The Miracle Tree Moringa oleifera:Natural Nutrition For The Tropics, Training Manual Church World Services, Dakar, Senegal.

Fuglie L.J (2001).Combatting Malnutrition with Moringa. Senegal: Church World Service,

Fung E.B, Samson-Fang L, Stallings V.A, (2002).Feeding dysfunction is associated with poor growth and health status in children with CP. J Am Diet Assoc.;102:361-373.

Ghai O.P, Gupta P, Paul V.K (2006). Essential Pediatrics, Adolescent Health and Development.Pediatrics; 6:66.

Gisel E.G, Patrick J: Identification of children with cerebral palsy unable to maintain a normal nutritional status. Lancet 1988; I: 283-6.

GOK, (2008). The Kenya National Technical Guidelines for Micronutrient Deficiency Control. Ministry of Public Health and Sanitation. Nairobi. Kenya.

Gopalan, C., Rama Sastri, B. V., & Balasubramanian, S. C. (2009). Nutritive value of Indian foods. Hyderabad, India: National Institute of Nutrition, Indian Council of Medical Research.

Gopalan C, Ramshashtri B .V, Balasubramanian S.C (1999). Nutritive value of Indian foods. Hyderabad, NIN, p.156

Gottlieb C.A, et al., (2009). Child disability screening, nutrition, and early learning in 18 countries with low and middle incomes: data from the third round of UNICEF's Multiple Indicator Cluster Survey (2005-06). Lancet374 (9704):p. 1831-9.

Grantham-McGregor S and Ani C (2001). "A Review of Studies on the Effect of Iron Deficiency on Cognitive Development in Children," Journal of Nutrition, Vol. 131,649S-666S

Grantham-McGregor, S. M., Powell, C. A., Walker, S. P., & Himes, J. H. (1991).Nutritional supplementation, psychosocial stimulation, and mental development of stunted children: the Jamaican Study. L ancet, 338, 1-5.

Henderson R.C, Grossberg RI, Matuszewski J, et al. (2007).Growth and nutritional status in residential center versus home-living children and adolescents with quadriplegic cerebral palsy. J.Pediatr. 151:161-166.

Henderson R.C, Kairalla J.A, Barrington J.W, et al. (2005).Longitudinal changes in bone density in children and adolescents with moderate to severe cerebral palsy. J Pediatr; 146:769-775.

Hillesund E, Skranes J, Trygg K, Bohmer T. (2007). Micronutrient status in children with cerebral palsy Acta Paediatra; 96: 1195-1198.

Himmelmann, K. Hagberg G, Uvebrant P. (2010). The changing panorama of cerebral palsy in Sweden. X. Prevalence and origin in the birth year period 1991 -1994; ACTA Paediatrica.99: 1337-1343.

Hirtz D, Thurman D.J, Gwinn-Hardy K, Mohamed M, Chaudhuri A.R, Zalutsky R .(2007). "How common are the "common" neurologic disorders?” Neurology 68 (5):326-337.

Hung J. W, Hsu T. J, Wu P. C, Leong C. P (2003). Risk factors of under nutrition in children with spastic cerebral palsy. Chang Gung Medical Journal, 26, 425-432.

Hustad K.C, Gorton K and Lee J. (2011). Classification of speech and language profile in 4years old children with cerebral palsy. National Institute of Health Public Access Author Manuscript, 53(6):1496-1513.

Ifeyinwa B.Okeke et al.,( 2010).Nutritional status of children with cerebral palsy in Enugu,Nigeria.European Journal of scientific research vol.39(4)505-513.

Ikekpeazu et.al., (2010).Serum vitamin A levels in children with protein energy malnutrition

Author(s): Vol. 14, No. 1

Jevsevar D.S, Karlin L.I (1993).The relationship between preoperative nutritional status and complications after an operation for scoliosis in patients who have cerebral palsy. J Bone Joint Surg Am.; 75:880-884

Kayi K.K (2013). A study on Moringa oleifera leaves as a supplement to West African weaning foods.Unpblished Thesis

Kennedy W.M (1990). An investigation of current practices in education of the physically handicapped in Kenya and their effects on curriculum development, examinations and methods of teaching. Unpublished manuscript: Kenya Institute of Education at Nairobi.

Kent Ruth (2013). "Chapter 38: Cerebral Palsy". In Barnes MP, Good DC. Handbook of Clinical eurology. 3 110. Elsevier. pp. 443-459.

Kerac M. et al., (2014).The interaction of malnutrition and neurologic disability in Africa. Semin Pediatr Neurol, 21(1): p. 42-9.

Kilpinen P, Pihko H, Vesander U, Paganus A, Ritanen U, et al: Insufficient energy and nutrient intake in children with motor disability. Acta Peediatrica 2009; 98: 1329-1333.

Kong C.K, Wong H.S.(2005).Weight-for-height values and limb anthropometric composition of tube-fed children with quadriplegic cerebral palsy. Pediatrics 116:e839-45.

Koriata N.S. (2012).Nutritional status of children with cerebral palsy attending Kenyatta National Hospital. Unpublished master's thesis.

Krick J, Murphy Miller P, Zeger S (1996). Pattern of Growth in Children with Cerebral Palsy J Am Diet Assoc.; 96:680-5.

Krick J, Murphy P.E, Markham J.F, Shapiro B.K (1992).A proposed formula for calculating energy needs of children with cerebral palsy. Dev Med Child Neuro1; 34:481-7.

Lewis D, Khoshoo V, Pencharz P.B, et al., (1994).Impact of nutritional rehabilitation on gastroesophageal reflux in neurologically impaired children. J Pediatr Surg.29:167-170.

Lim T.K (2012). "Moringa Oleifera." Edible Medicinal and Non-Medicinal Plants: 453-485.

Liu J, Raine A, Venables P.H, et al., (2003).Malnutrition at age 3 years and lower cognitive ability at age 11 years: independence from psychosocial adversity. Arch Pediatr; Adolesc Med.; 157:593-600.

Machin D, Campbell M.J and Walters S.J (2007). Medical statistics: A textbook for health sciences (4th edition). West Sussex: John Wiley and Sons.

Marcu G.M (2005),"Miracle Tree" KOS Health Publications, 466 Foothill Blvd.

Maulik, P.K. Darmstadt, G.L (2007). Childhood Disability in Low- and Middle-Income Countries: Overview of Screening, Prevention, Services, Legislation, and Epidemiology Pediatrics 120, SI-S55.

Mbithi-Mwikya S, Ooghe W, Van Camp J, Nagundi D, Huyghebaert A (2000). Amino acid profile after sprouting, Autoclaving and lactic acid fermentation of finger millet (Elusine coracana) and kidney beans (*Phaseolus vulgaris* L.) J. Agric. Food Chem.48 (8): 3081-3085.

Munk D.D (1994). Behavioral assessment of feeding problems of individuals with severe disabilities. J Applied Behavior Analysis 27: 241-250.

Nandy S, Irving M, Gordon D, Subramanian S.V, Smith G.D (2005). Poverty, child undernutrition and morbidity: New evidence from India. Bull World Organ, 83: 210-216.

Ngare, D., Mutunga, J., and Njoroge, E. (2000). Vitamin A Deficiency in Pre-school

Children in Kenya. East Africa Medical Journal. Volume 77.

Olson, M. E. Carlquist, S. (2001). "Stem and root anatomical correlations with life form diversity,ecology, and systematics in Moringa (Moringaceae)". Botanical Journal of the Linnean Society 135 (4).

Oskoui, M, Coutinho F, Dykeman J, Jetté N, Pringsheim T (2013). "An update on the prevalence of cerebral palsy: a systematic review and meta-analysis." Developmental medicine and child neurology 55 (6): 509-19.

Pencharz P.B. (2010).Protein and energy requiements for 'optimal' catch-up growth. Eur J Clin Nutr; 6S5-S7.

Peter K.V (2008). Underutilized and Underexploited Horticultural Crops: Volume 4. New India Publishing. p. 112. ISBN 81-89422-90-1

Price M.L (2002).The Moringa Tree. Educational Concerns for Hunger Organization (ECHO) Technical Note. 1985, revised May.

Reddihough D.S, Baikie G & Walstab J.E (2001) Cerebral Palsy in Victoria, Australia: Mortality and causes of death. J Paediatr Child Health 37, 183-186.

Reddihough D.S, Collins, K.J (2003). The epidemiology and causes of cerebral palsy. Australian Journal of Physiotherapy. 49(1): 7-12.

Reilly S, Skuse D, & Poblete, X (1996). Prevalence of feeding problems and oral motor dysfunction in children with cerebral palsy: a community survey. Journal of Pediatrics, 129, 877-882.

Rogerson R, Gallagher M, Beebe A (2000). Flexible tape is an appropriate tool for knee height measurement and stature estimation in adults with developmental disabilities. J Am Diet Assoc. 100:105-7.

Rosenbaum P, Paneth N, Leviton A, Goldstein M, Bax M, Damiano D, Dan B, Jacobsson, B (2007). "A report: The definition and classification of cerebral palsy"Developmental Medicine & Child Neurology 49: 8-14.

Samson-Fang L, Stevenson R.D (1998). Linear growth velocity in children with cerebral palsy. Dev Med Child Neurol.; 40:689-692

Sauveur, A.D. and Hartout, G. (2001). Moringa culture and economy in Niger.

Schmidt L.H, & Mwaura, L (2010). Moringa oleifera Lam. Seed leaflet

Sehemji J.K. (1993). National Food Composition Tables and Planning for Satisfactory Diets in Kenya Ministry of Health and National Public Services.

Sena, L.P, (1998). "Analysis of nutritional components of eight famine foods of the Republic of Niger." Plant Foods for Human Nutrition: 17-30.

Shahnawaz. M, Sheikh, A. Sand Nizamani, S. M. (2009). Determination of Nutritive Values of Jamun Fruit *(Eugenia jambolana)* Products. Pakistan Journal of Nutrition 8 (8): 1275-1280, 2009 ISSN 1680-5194. Asian Network for Scientific Information.

Singh, P. & Raghuvanshi, R. S. (2012). Finger millet for food and nutritional security. African Journal of Food Science, 6**,** 77-84

Sjakti H, Ambara D, Rusli S, Luh K, Wahyuni I (2008). Feeding difficulties in children with cerebral palsy.Paediatrica Indonesiana vol 48 No.4.

Smart J (1993).Malnutrition, learning, and behavior: 25 years on from the MIT symposium. Proc Nutr Soc.; 52:189-199.

Spender Q.W, Cronk C.E, Charney E.B, Stallings V.A (1989). Assessment of linear growth of children with cerebral palsy: use of alternative measures to height or Med Child Neurol. 31:206-14 Length. Dev.

SPSS (2008). Statistical Package for Social Science, Computer Software, IBM, SPSS Ver.16.0 in 2008., SPSS Company, London, UK.

Stallings V.A, Charney EB, Davies J.C, et al., (1993).Nutritional status and growth of children with diplegic or hemiplegic cerebral palsy. Dev Med Child Neurol.; 35:997-1006.

Stallings V.A, Cronk CE, Zemel BS, et al. (1995).Body composition in children with spastic quadriplegic cerebral palsy. J Pediatr; 126:833-839

Stallings V.A, Zemel B.S, Davies J.C, et al., (1996).Energy expenditure of children and adolescents with severe disabilities: a cerebral palsy model. Am J Clin Nutr.; 64:627-634.

Stevenson R.D, Conaway M, Chumlea W.C, et al., ( 2006).Growth and health in children with moderate-to-severe cerebral palsy. Pediatrics. 118:1010-1018.

Stevenson R.D, Hayes R.P, Cater L.V, et al., (1994).Clinical correlates of linear growth in children with cerebral palsy. Dev Med Child Neurol.; 36:135-142.

Stevenson R.D. (1995).Use of Segmental Measures to Estimate Stature in Children with Cerebral Palsy. Arch Pediatr Adolesc Med.; 149:658-62.

Strauss D, Shavelle R, Reynolds R, et al., (2007) Survival in cerebral palsy in the last 20 years: signs of improvement? Dev Med Child Neurol.; 49:86-92.

Strauss D, Shavelle, R. M, & Anderson, T.W (1998). Life expectancy of children with cerebral palsy. Pediatric Neurology, 18, 143-149.

Sullivan P.B, Juszack E, Lambert B.R, Rose M, Ford-Adams M.E & Johnson A (2002) .Impact of feeding problems on nutritional intake and growth: Oxford feeding study II. Dev Med Child Neurol; 44, 461-467.

The Government of Kenya, Ministry of Planning and National Development (2007). Kenya Vision 2030.

The Government of the Republic of Kenya (2001).The Children Act, (Act No. 8 of 2001)

The Government of the Republic of Kenya (2003). The Persons with Disabilities Act, 2003

The Government of the Republic of Kenya (2011). National report: Kenya’s initial report submitted under article 35(1) of the United Nations Convention on the Rights of Persons with Disabilities.

Thommessen M, Kase B.F, Riis G & Heiberg A (1991).The impact of feeding problems on growth and energy intake in children with cerebral palsy. Eur J Clin Health 45, 479-487.

United Nations (1986). How to Weight and Measure Children: Assessing the Nutritional Status of Young Children in Household Surveys. New York: United Nations.

United Nations, Convention on the rights of persons with disabilities. (2006).United Nations: New York.

UNHCRIUNICEF/WFP/WHO (2002). Guidelines. Rome, World Food Programme

UNICEF, (2003). The State of the World‘s Children 2004. UNICEF. New York USA. pg 19.

Viteri F.E, Walker A.W, Durie P.R, et al., (1991). Protein energy malnutrition. In: Editor’s Pediatric gastrointestinal diseases. B.C. Decker; Philadelphia: pp. 1596–1611.

Wagner J, and Stanton T (2009). Formulating Rations with the Pearson Square. Colorado State University. 1:618. http://www.ext.colostate.edu/ UBS/LIVESTK/ 01618.html

Walker J.L, Bell K.L, Boyd R.N, Davies P.S. (2012).Energy requirements in preschool-age children with cerebral palsy. Am J Clin Nutr; 96: 1309-1315.

WHO (2011).World Report on Disability, Geneva: World Health Organisation.

World Health Organization (2009). Global database on child growth and malnutrition. Geneva. ([www.who.int/nutgrowthdb/database/en](http://www.who.int/nutgrowthdb/database/en)).

WHO (2006).Multicentre Growth Reference Study Group WHO Child Growth Standards based on Length/height, weight and age. Acta Paediatr Suppl.; 450:76-85.

WHO (2000). Comparartive Quantification of Health Risk, Global and Regional Burden of Disease Attributable to Selected Major Risks Factors. WHO Cataloguing. Volume 1. Geneva

WHO (1999). Management of severe malnutrition: a manual for physicians and other senior health workers. Geneva: World Health Organisation.

Yeargin-Allsop M, Braun KVN, Doernberg NS, et.al.,.( 2008).Prevalence of cerebral palsy in 8-year old children in three areas of the United States in 2002: a multisite collaboration. Pediatrics; 121:547-554.

Young V.R and Pellett P.L (1994). Plant proteins in relation to human protein and amino acid nutrition. Am. J. Clin. Nutr. 59: 1203S-1212S.

Yousafzai, A.K., S. Filteau, and S. Wirz,( 2003). Feeding difficulties in disabled children leads to malnutrition: experience in an Indian slum. The British journal of nutrition, 90(6): p; 1097-106.

Zongo, U, Zoungrana, S.L, Savadogo, A, Traore, A.S (2013). Nutritional and clinical rehabilitation of severely malnourished children with Moringa oleifera Lam. Leaf powder in Ouagadougou (Burkina Faso). Food Nutr. Sci., 4: 991-997.

**Appendix A: Informed consent and introduction form**

**
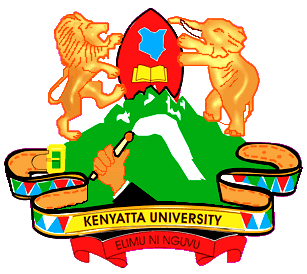
**

**KENYATTA UNIVERSITY**

**ETHICS REVIEW COMMITTE**

**Informed Consent**

My name is **Janet Kajuju Malla.** I am a Ph.D student from Kenyatta University. I am conducting a study on “Effect of *Moringa oleifera* Consumption on Protein and Vitamin A Status of Children with Cerebral Palsy in Nairobi, Kenya: A Randomized Controlled Trial” The information will be used by the Ministry of Medical Services and Ministry of Public Health and sanitation in managing malnutrition among children with Cerebral Palsy (CP) hence ensure adequate growth and improved quality life.

**Procedures to be followed**

Participation in this study will require that I ask you some questions and I also examine your child by taking anthropometric measurements. Blood samples will be drawn from your child by a certified phlebotomist under the standard operational procedures. Five milliliters of blood will be collected through venipuncture of a peripheral vein using disposable syringes. The syringes will safely be disposed off into the syringe disposal container. The specimen will be put in portable freezers for transportation to the laboratory for biochemical analysis of serum albumin and retinol levels. Your child may experience some pain during the procedure which disappears after but if the pain persists you will be referred for medical treatment. Your child will receive one cup of porridge at 10.00 o’clock daily for three months. In case of any toxicities, the participants will be withdrawn from the study and referred to a medical centre

I will record the information from you and your child in a questionnaire.

You have the right to refuse participation in this study. Your decision will not change the care your child will receive from the centre today or at any other time.

Please remember the participation in this study is voluntary. You may ask questions related to the study at any time.

You may refuse to respond to any questions and you may stop an interview at any time. You may also stop being in the study at any time without any consequences to the services you receive from this centre or any other organization now or in the future.

**Discomforts and Risks**

Some of the questions you will be asked are on intimate subject and may be embarrassing or make you uncomfortable. If this happens, you may refuse to answer these questions if you so choose. You may also stop the interview at any time. The interview may add approximately half an hour to the time you wait before you receive your routine services.

**Benefits**

If you and your child participate in this study, your child will benefit from the nutrition support given in terms of porridge provided and if your child is found to be malnourished, you will be referred to a health facility for treatment. You will also help us to learn how to use locally available indigenous foods in preventing and managing malnutrition among children, especially those with disabilities like CP.

**Reward**

You will not receive any payment for participating in the discussions. You will also not be charged any fee for participating in the discussions.

**Confidentiality**

The interviews and examinations will be conducted in a private setting within the centre. Your name will not be recorded on the questionnaire. The questionnaires will be kept in a locked cabinet for safe keeping. Everything will be kept private. Professionalism will be highly upheld to in store confidence among the participants and the community.

**Contact Information**

If you have any questions you may contact Dr. Sophie Ochola on 0721449803 or Dr. Irene Ogada on 0723955466 or the Kenyatta University Ethical Review Committee Secretariat on [chairman.kuerc@ku.ac.ke](mailto:chairman.kuerc@ku.ac.ke), [secretary.kuerc@ku.ac.ke](mailto:secretary.kuerc@ku.ac.ke), [secretariat.kuerc@ku.ac.ke](mailto:secretariat.kuerc@ku.ac.ke)

**Participant’s statement**

The above information regarding my participation in the study is clear to me. I have been given a chance to ask questions and my questions have been answered to my satisfaction. My participation in this study is entirely voluntary. I understand that my records will be kept private and that I can leave the study at any time. I understand that my child will still get the same care whether I decide to leave the study or not and my decision will not change the care that my child will receive from the centre today or at any other time.

Name of Participant…………………………………………………………………

Signature or Thumbprint Date

**Investigators statement**

I, the undersigned, have explained to the volunteer in a language s/he understands, the procedures to be followed in the study and the risks and benefits involved

Name of Interviewer………………………………………………………

Signature or Thumbprint Date

## Appendix B1: Preparation of M. oilefera leaf powder

The stalks holding the leaves of *M. oleifera* will be cut from the tree and brought to the laboratory, where the leaves will be removed from the stalks. The leaves will then be immersed in clean water in troughs and shaken in order to remove dirt and impurities on the leaf surfaces. They will be washed again in 1% saline solution (NaCl) for about three to five minutes (3-5minutes), to remove microbes logged on the leaves. It is necessary to rinse again in clean water to remove the saline content before proceeding to drain the water from the leaves. The troughs will be drained after each batch and clean water used for the washing of each successive batch (Armelle and Melanie, 2010; Amaglo et al., 2007). The washed leaves will then be steam blanched for 3 minutes. Blanching is necessary for preserving the colour, flavour and to inactivate enzymes to prevent nutrient losses (Kendall, 2003). The leaves will then be cooled in ice water for 5 minutes to prevent continued heat damage of nutrients and then spread out on racks for 20 min to drain out water. They will then be solar dried on racks. The dried leaves will be milled using a kitchen blender, packaged in a translucent or coloured polythene bag and kept in a plastic container with cover and stored at room temperature for chemical analysis.

## Appendix B2: Fermentation of finger millet

Fermentation will involve soaking the grain in water for a period of 48 hours at room temperature. The water will be drained and the grains fermented for 48 hours in air tight containers. At the completion of fermentation, the pH will be determined. The grains will then be dried to a moisture content of 12.0%. The millet grains will then be extruded at 115 °C to attain moisture content of 9% then milled into flour. Fermentation improves palatability and bio-availability of nutrients by reducing the anti-nutrients found in cereals.

## Appendix C: Proximate analysis

Proximate analysis will be used to determine the moisture, ash, fat, protein, fibre. The standard method of Association of Official Analytical Chemists, (AOAC, 2005) will be applied. Carbohydrate content will be obtained by difference method as described by FAO (2010), Shahnawaz et al. (2009) and James (1995). Crude protein content will be determined using the micro-Kjeldahl method as described by Pearson (1976). Crude fat will be estimated by employing solvent extraction using a Soxhlet extraction unit (AOAC, 2005). Ash contents will be determined according to AOAC (Association of Analytical Chemists) number 923.03 (AOAC 2005). Anti-nutrients will be determined using Swain (1979) method to determine tannins, Wheeler and Ferrel (1971) method for phytate determination, (AOAC, 2005) method for oxalate determination. The content of serum albumin, retinol and β – carotene will be established using UV–VIS Spectrophotometry method. Procedures are described below.

1. **Determination of moisture content**

One gram of sample will be weighed in a pre-weighed crucible and placed in an oven (105°C) for 3hrs, cooled in a dessicator and reweighed. The percent moisture will be calculated as follows:


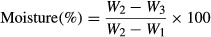


Where: *W*1 is the weight of the crucible, *W*2 is the total weight of the crucible and sample after drying at 105°C and sample, and *W*3 is the total weight of the crucible and the dried sample after cooling in airtight desiccators.

1. **Determination of crude protein**

Crude protein content will be determined using the micro-Kjeldahl method as described by Pearson (1976). 0.5g of the sample will be weighed into a digestion flask. About 0.3g of the catalyst will be added to the flask containing the sample, followed by the addition of 20ml of concentrated sulphuric acid. (H_2_SO_4_) The mixture will then be digested until it is coulourless. The digested mixture will then be cooled to room temperature and then diluted with 50ml distilled water. The digested mixture will then be neutralized by adding 90ml of sodium hydroxide solution (32%). The neutralized solution will then be distilled into a receiving flask containing 60ml of boric acid solution(4%) containing Bromocresol and methyl red indicators. The distillate will then be titrated using standard HCL solution (0.02M).A blank determination (without sample) will also be carried out.


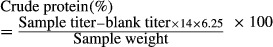


Where: 14 is the molecular weight of nitrogen and 6.25 is the protein conversion factor factor.

1. **Determination of crude fat**

Crude fat will be estimated by employing solvent extraction using a Soxhlet extraction unit (AOAC, 2005).

One gram sample will be weighed into an extraction thimble and covered with absorbent cotton. 50 ml solvent (petroleum ether) will be added to a pre-weighed flask. Both thimble and flask will be attached to the extraction unit .The sample will then be subjected to extraction with solvent for 30 minutes followed by rinsing for 1.5 hrs. Afterwards, the solvent will be vacuum evaporated from the flask to the condensing column. Extracted fat in the flask will be placed in an oven at 110°C for 1 hr to evaporate the solvent. The weight of extracted lipid will be determined by subtracting the weight of the empty flask from the total weight of flask + fat after drying. Crude fat will be calculated using the following formula:

Crude fat (%) = (Extracted fat / Sample weight) x 100

1. **Determination of ash**

Ash content will be determined according to AOAC (Association of Analytical Chemists) method number 923.03 (AOAC 2005). Two grams of sample will be added into a pre-weighed crucible and incinerated in muffle furnace at 600°C. Ash content will be determined using the following formula


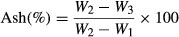


Where: *W*1 is the weight of cleaned, dried, and cooled crucible, *W*2 the weight of the crucible and sample before incinerating at 600°C and *W*3 the weight of the crucible and incinerated sample after cooling in a dessicator.

1. **Determination of carbohydrate**

The carbohydrate content will be determined by difference according to FAO (2010), Shahnawaz et al. (2009), and James (1995), that is, addition of all the percentages of moisture, fat, crude protein and ash, and subtracted from 100%. This gives the amount of nitrogen-free extract otherwise known as carbohydrate.

%Carbohydrate =100 – (% Moisture +% Fat%+% Ash+%Crude protein)

1. **Determination of total phenolic compounds**

The samples (100 g) will be extracted by mixing with 250 ml of methanol for 3 hrs. The extracted samples will then be filtered through Whatman No. 1 filter paper, the residue washed with 100 ml methanol, and the extracts cooled and evaporated to dryness under vacuum using a rotary evaporator. The residues will be dissolved with 10 ml of methanol and used for determination of total phenolic compounds. The absorbances of the phenolic compound standard solutions as well as samples will be obtained using UV-VIS spectrophotometer at a wavelength of 760 nm.

1. **Determination of tannin content**

Tannin content of the flour samples will be determined using the methods described by Swain (1979). The sample (0.2 g) will be placed in a 50-ml beaker followed by addition of 20 ml of 50% methanol. The mixture will then be homogenized, placed in a water bath at 77–80°C for 1 hr, and the contents stirred with a glass rod to prevent lumping. The mixture will then be filtered using a double-layered Whatman No. 1 filter paper into a 100-ml volumetric flask using 50% methanol to rinse. The filtrate will then be made up to mark with distilled water and thoroughly mixed. One milliliter of the sample extract will be pipetted into a 50-ml volumetric flask, and 20 ml distilled water, 2.5 ml Folin-Denis reagent, and 10 ml of 17% Na_2_CO_3_ will be added and mixed. The mixture will then be made up to mark with distilled water, thoroughly mixed, and allowed to stand for 20 min until a bluish-green coloration develops. Standard tannic acid solutions in the range of 0–10 ppm will be treated similarly as the 1 ml sample above and will be used to obtain a standard curve. The absorbances of the tannic acid standard solutions as well as samples will be obtained using UV-VIS spectrophotometer at a wavelength of 760 nm. The concentration of tannins from the sample will be determined through extrapolation of the standard curve.

1. **Determination of phytic acid**

An indirect colorimetric method of Wheeler and Ferrel (1971) will be used for phytate content determination. Five grams of the sample will be extracted with 3% trichloro acetic acid. The phytates will be precipitated as ferric phytate and converted to ferric hydroxide and soluble sodium phytate by adding sodium hydroxide. The precipitate will be dissolved in hot 3.2 N HNO and the absorbance read immediately at 480 nm. The standard solution will be prepared from Fe (NO_3_)_3_ and the iron content will be extrapolated from a Fe (NO)_3_ standard curve. The phytate concentration will calculated from the iron concentration determined for the samples, assuming a 4:6 iron:phosphorus molecular ratio.

1. **Determination of oxalate content**

Oxalate will be determined according to AOAC (2005) method. One gram of the sample will be weighed in a 100-ml conical flask. Seventy-five milliliters of H_2_SO_4_ (3M) will be added and the solution stirred intermittently with a magnetic stirrer for about 1 hr, followed by filtering using Whatman No. 1 filter paper. The sample filtrate /extract (25 ml) will be collected and titrated against hot (80–90°C) 0.1 N KMnO_4_ solution to the point where a faint pink color appear and persist for at least 30 sec. The concentration of oxalate in each sample will be obtained from the calculation: 1 ml 0.1 permanganate = 0.006303 g oxalate.

1. **Determination of β -carotene content**

The content β - carotene will be established using UV–VIS Spectrophotoscopy method. To extract β - carotene, 50 mls of acetone–hexane mixture containing 0.1% BHT will be added to 5 g sample and the mixture shaken for 10 minutes, centrifuged and decanted to a separating funnel. The supernatant will be saponified by adding 25 mls of 0.5M methanolic potassium hydroxide, then shaken and allowed to settle for 30 minutes and then washed with 100 mls portions of distilled water. The aqueous layer will be discarded continuously. The extract will then be dried by filtering over anhydrous sodium sulphate. The filtrate will be concentrated in a rotary evaporator at 45°C and reconstituted in methanol to 50 mls. Different concentrations of standard solution will be prepared using 95% UV β – carotene. A stock solution of (100 μg/ml) will be made by dissolving 0.01 g of β - carotene standard into 10 mls hexane, which will then increased to 100 mls. The working standard solution will be used to prepare standard solutions of various concentrations. The absorbance (A) of each concentration will be measured using the UV–Vis Spectroscopy at a wavelength of 545nm (Gupta et al., 2005).

## Appendix D: Nutrient profile form

1. **Nutritional composition of dried *M. oleifera* leaf powder and millet flour**

| **Parameter** | **Percent (%)** |
| --- | --- |
| Moisture |  |
| Fat |  |
| Crude Protein |  |
| Crude Fibre |  |
| Ash |  |
| Carbohydrate |  |
| Vitamin A (carotenoids) |  |

1. **Anti-nutritional factors in *M. oleifera*** **leaf powder and millet flour**

| **Factor** | **Quantity** |
| --- | --- |
| Total phenols (g/kg) |  |
| Tannin (g/kg) |  |
| Phytate (g/kg) |  |
| Oxalate (g/kg) |  |

## Appendix E: Acceptability of the M. oleifera fortified millet porridge by the caregiver’s of children

Tick against the appropriate response

| **Quality Attributes** | **Responses** | | | | |
| --- | --- | --- | --- | --- | --- |
|  | Extremely Like (5) | moderately like (4) | neither like nor dislike (3) | moderately dislike (2) | Extremely dislike (1) |
| Colour |  |  |  |  |  |
| Texture |  |  |  |  |  |
| Taste |  |  |  |  |  |
| Overall Acceptability |  |  |  |  |  |

## Appendix F1: Laboratory request form

Date……………………………………………

Serial No…………………File No……………

| S/No | Serum albumin levels  ( μmol/L) | serum retinol levels  ( μmol/L) |
| --- | --- | --- |
|  |  |  |

**Phlebotomy:**

Done by (Name) _______________________________________

Date ________________________________________________

Sign ________________________________________________

**Analysis**

Done by (Name) _______________________________________

Date ________________________________________________

Sign ______________________________________________

## Appendix F2: Biochemical determination of retinol

Retinol is extracted from serum with ethanol containing butyl-hydroxytoluene (BHT). Retinol is determined by HPLC with spectrofiuorimetric detection (λex 340 nm; λcm 460 nm). Duker et al., (2009).

## Appendix F3: Biochemical determination of serum albumin

Bromocresol green (BCG) ASSAY method will be is used. Albumin in the sample reacts with bromocresol green (BCG) in acid medium forming a coloured complex that can be measured by a colorimeter. One ml reagent will be pipetted into three test tubes which are labeled as blank, sample and standard. 0.02 ml of sample will be added to the sample test tube, and 0.2 ml of standard will be added to standard tube, 0.02 ml distilled water will added to blank test tube. Contents of each tube will be mixed thoroughly and allowed to stand for 10 minutes. The absorbances (Abs) of the standard and the samples will be read in spectrophotometer at 545 nm against the blank.

The albumin concentration in the samples will be calculated using the following general formula:

Albumin (g/dl) = Abs sample X Concentration of standard.

Abs standard

**Appendix F4: Nutritional value of *M. oleifera* leaf powder per 100 grams of edible portion:**

|  | Moringa fresh Leaves | Moringa Leaf Powder |
| --- | --- | --- |
| Moisture (%) | 75.0 | 7.5 |
| Calories | 92.0 | 205.0 |
| Protein (g) | 6.7 | 27.1 |
| Fat (g) | 1.7 | 2.3 |
| Carbohydrate (g) | 13.4 | 38.2 |
| Fiber (g) | 0.9 | 19.2 |
| Minerals (g) | 2.3 | - |
| Ca (mg) | 440.0 | 2,003.0 |
| Mg (mg) | 24.0 | 368.0 |
| P (mg) | 70.0 | 204.0 |
| K (mg) | 259.0 | 1,324.0 |
| Cu (mg) | 1.1 | 0.6 |
| Fe (mg) | 7 | 28.2 |
| S (mg) | 137.0 | 870.0 |
| Oxalic acid (mg) | 101.0 | 0.0 |
| Vitamin A - B carotene (mg) | 6.8 | 16.3 |
| Vitamin B -choline (mg) | 423.0 | - |
| Vitamin B1 -thiamin (mg) | 0.21 | 2.6 |
| Vitamin B2 -riboflavin (mg) | 0.05 | 20.5 |
| Vitamin B3 -nicotinic acid (mg) | 0.8 | 8.2 |
| Vitamin C -ascorbic acid (mg) | 220.0 | 17.3 |
| Vitamin E -tocopherol acetate (mg) | - | 113.0 |
| Arginine (g/16g N) | 6.0 | 0.0 |
| Histidine (g/16g N) | 2.1 | 0.0 |
| Lysine (g/16g N) | 4.3 | 0.0 |
| Tryptophan (g/16g N) | 1.9 | 0.0 |
| Phenylanaline (g/16g N) | 6.4 | 0.0 |
| Methionine (g/16g N) | 2.0 | 0.0 |
| Threonine (g/16g N) | 4.9 | 0.0 |
| Leucine (g/16g N) | 9.3 | 0.0 |
| Isoleucine (g/16g N) | 6.3 | 0.0 |
| Valine (g/16g N) | 7.1 | 0.0 |

## Appendix G: Questionnaires

**Appendix G1: Questionnaire for caregivers**

**Social economic and demographic data of caregivers**

Fill in the spaces or tick where necessary

1. Age in complete years of caregiver
   1. Mother….......... (Yrs)
   2. Father…........... (Yrs)
2. Sex Male (1) Female (2) of caregiver
3. Household head
   1. Female headed….............
   2. Male headed…………….
4. Marital status of caregiver
   1. Married
   2. Single
   3. Divorced
   4. Cohabit
5. Education level of caregiver to completion
6. University level
7. College level
8. Secondary level
9. Primary level
10. No formal education
11. Occupation of caregiver
    1. Paid employment
    2. Self employment
    3. Casual labour
    4. Others (Specify)….......
12. Number of children in the family………………

**Appendix G2: Child’s information**

1. Age in complete Years ………….
2. Sex
3. Male
4. Female
5. Has this child been sick in the past seven days?
6. Yes
7. No

If yes what is the illness………………………..

1. Does the child have difficulties in chewing?
2. A lot of difficulty
3. Sometimes
4. Never
5. Does the child vomit at mealtimes?
   1. A lot of vomitting
   2. Sometimes
   3. Never
6. Does the child feed by herself/himself?
7. Yes
8. No
9. Sometimes
10. Is the child currently on medication?( a) Yes (b) No
11. If yes, is it (a) long term? (b) Short term?
12. Does the child experience any side effects to the medication above?
13. Yes b) No

10. At what age was the child diagonised with CP?

**Appendix G3: Dietary intake of the child**

1. **Food frequency table**

How many times did the child consume the foods listed below in the last 7 days? Tick appropriately

|  | **Frequency of consumption in the last one week (Tick where appropriate)** | | | | | | | |
| --- | --- | --- | --- | --- | --- | --- | --- | --- |
|  | **Once** | **2 times** | **3 times** | **4 times** | **5 times** | **6 times** | **Daily** | **Never** |
| **Proteins** | | | | | | | | |
| Fish |  |  |  |  |  |  |  |  |
| Eggs |  |  |  |  |  |  |  |  |
| Milk |  |  |  |  |  |  |  |  |
| Chicken |  |  |  |  |  |  |  |  |
| Beef |  |  |  |  |  |  |  |  |
| Beans |  |  |  |  |  |  |  |  |
| **Starches:** | | | | | | | | |
| Chips |  |  |  |  |  |  |  |  |
| Ugali |  |  |  |  |  |  |  |  |
| Rice |  |  |  |  |  |  |  |  |
| Chapati |  |  |  |  |  |  |  |  |
| Yams |  |  |  |  |  |  |  |  |
| Arrowroots |  |  |  |  |  |  |  |  |
| Mashed potatoes |  |  |  |  |  |  |  |  |
| **Fats:** | | | | | | | | |
| Butter/margarine |  |  |  |  |  |  |  |  |
| Cream |  |  |  |  |  |  |  |  |
| Peanut butter |  |  |  |  |  |  |  |  |
| Cheese |  |  |  |  |  |  |  |  |
| **Fruits:** | | | | | | | | |
| Oranges |  |  |  |  |  |  |  |  |
| Bananas |  |  |  |  |  |  |  |  |
| Avocado |  |  |  |  |  |  |  |  |
| Pawpaw |  |  |  |  |  |  |  |  |
| Passion |  |  |  |  |  |  |  |  |
| Pineapple |  |  |  |  |  |  |  |  |
| **Vegetables:** |  |  |  |  |  |  |  |  |
| Cabbage |  |  |  |  |  |  |  |  |
| Carrots |  |  |  |  |  |  |  |  |
| Spinach |  |  |  |  |  |  |  |  |
| Sukuma wiki |  |  |  |  |  |  |  |  |
| Cow pea leaves |  |  |  |  |  |  |  |  |
| Tomatoes |  |  |  |  |  |  |  |  |
| Other vegetables |  |  |  |  |  |  |  |  |

1. **24-hour recall**

**TICK THE DAY OF THE WEEK WHICH YOU ARE RECALLING** (IT SHOULD BE THE DAY BEFORE THE INTERVIEW)

| Monday | Tuesday | Wednesday | Thursday | Friday | Saturday | Sunday |
| --- | --- | --- | --- | --- | --- | --- |

**Step 1**: Please think back to when the child woke up yesterday morning to the time he/she went to sleep in the evening. Now, I want you to try and remember what the child ate or drank yesterday from the moment he/she got up until they went to sleep again last night. Run through the whole day in your mind and try to remember everything that the child ate or drank. (THE INTERVIEWER MUST GIVE THE RESPONDENT A LITTLE TIME TO DO THIS). Now I would like to you tell me what the child ate and drank in the morning after he/she got up. AFTER THE PARTICIPANT MENTIONS AN ITEM, THE INTERVIEWER SHOULD PROMPT THE RESPONDENT BY SAYING “AND THEN?”

**ENTER THE INFORMATION IN COLUMN 1)**

| **STEP 1:** Food/drink eaten/drank during the day | **STEP 2:** Forgotten foods (PROMPTED) |
| --- | --- |
|  |  |
|  |  |
|  |  |
|  |  |
|  |  |

**STEP 2**: NOW ASK THE FOLLOWING QUESTIONS ON FORGOTTEN FOODS AND ENTER THEM IN COLUMN 2.

*Did the child have any cold drinks or soda yesterday?*

*Did the child have any sweets and or chocolate yesterday?*

*Did the child have any cake and or cookies yesterday?*

*Did the child have any snacks like chips, crisps, yesterday?*

*Did the child have any (other) fruit yesterday?*

*Did the child have any (other) vegetable yesterday?*

*Did the child have any bread yesterday? 144*

*Did the child have anything else yesterday?*

Q. What did the child eat/ drank yesterday; was it same as, more than or less than usual? (MARK **X** WHERE APPROPRIATE)

|  | Same as usual |  | More than usual |  | Less than usual |  |
| --- | --- | --- | --- | --- | --- | --- |
|  |  |  |  |  |  |  |

If more or less than usual, explain why (circle appropriately)

1. Celebration

2. Religious activity

3. Little food in the household

**4.** Other (specify) _______________________________

**Step 3: *“Now I am going to ask you more about each food or drink that you ate/drank yesterday”****.*

*START WITH THE 1ST ITEM REPORTED IN TABLE 1. TRANSFER THIS ITEM TO THE COLUMN 3 IN THE TABLE BELOW.*

*ASK “At what time was the item 1 eaten?”REPORT THE TIME IN COLUMN 1. DO NOT SPEND MUCH TIME IN GETTING THE EXACT HOUR.*

*ASK “for what meal was the item 1 eaten? INDICATE FOR WHAT MEAL ITEM 1 WAS EATEN AND REPORT IT IN COLUMN 2.’*

***Step 4 “Now I want you to tell me more about this food item….”***

THIS INCLUDES A DESCRIPTION OF THE FOOD AS WELL AS THE PREPARATION. **ENTER THIS INFORMATION IN COLUMN 4).** *“Now I want you to tell me more about this food item…….*

THIS INCLUDES A DETAILED DESCRIPTION OF THE FOOD (BRAND NAME, IF UNPROCESSED, SEMI-PROCESSED OR FULLY PROCESSED SIZE, ETC), THE AMOUNT PREPARED AND THE METHOD OF PREPARATION. ENTER THIS INFORMATION IN COLUMN 4.

USE STANDARD HOUSEHOLD MEASURES AND WEIGHTS TO DETERMINE AMOUNTS OF INGREDIENTS USED. INDICATE IF FOOD WAS PURCHASED ALREADY COOKED FROM THE STREETS BY INCLUDING THE FOLLOWING TEXT “STREET FOOD”NEXT TO THE ITEM

**S*tep 5. “****N****ow we are going to find out how much of this item was eaten/drank”***

*INTERVIEWER AND RESPONDENT USE HOUSEHOLD MEASURES AND WEIGHING EQUIPMENT TO DETERMINE HOUSEHOLD PORTION SIZES.*

THIS PROCESS IS REPEATED FOR EACH FOOD ITEM THAT WAS ENTERED ON FORM 1).

**STEP 4: RECORDING SHEET FOR INFORMATION COLLECTED IN STEP 3 OF THE 24-HOUR RECALL**

| 1 | 2 | | 3 | | | 4 | | | 5 | |  |
| --- | --- | --- | --- | --- | --- | --- | --- | --- | --- | --- | --- |
| Item carried from step 1 and 2 | Time | | Meal 1=breakfast2=lunch3=dinner4=snack5=other | | | Detailed description of the item before consumption (ingredients, quantities and cooking method) | | | Amount of food eaten | |  |
| description of ingredients (*Include* •*brand name,* • *if unprocessed, semi-processed and fully processed,* •*large, medium, small* | Quantity of ingredients | | Cooking method | | | Total amount cooked | | | Total amount eaten | |  |
| *description of ingredients in household measures* | *Weight (grams)* | *In HH measures* | |  | *Weight (grams)* | | *In HH measures* | *Weight*  *(grams* | | *In HH measures* | |

**Appendix G4: Child’s nutritional s**tatus

| **Parameter** | **Initial** | **Month 1** | **Month 2** | **Month 3** |
| --- | --- | --- | --- | --- |
| **Weight (Kg)**  1^St^ reading |  |  |  |  |
| 2^nd^ reading |  |  |  |  |
| Average |  |  |  |  |
| **MUAC (cm)**  1^St^ reading |  |  |  |  |
| 2^nd^ reading |  |  |  |  |
| Average |  |  |  |  |

**Appendix G5: KII guide**

1. How many children are enrolled at the centre?
2. .Out of these how many have special needs. Specify the conditions.
3. How is the centre managed in terms of finances? Is there support from the government, NGO’s, or parents?
4. What criteria do you use in recruitment of the children into the centre?
5. What challenges do you encounter in the management the special children?
6. What are the services offered at the centre?
7. Have these services been of benefit to the children?

## Appendix H: Morbidity

1. Has the child been sick in the last TWO WEEKS?

2. If yes, state how many times

3. Indicate the diseases, duration and the clinical manifestation

| Disease | Number of episodes | Duration in days | Clinical manifestations* |
| --- | --- | --- | --- |
|  |  |  |  |

***** 1. Epileptic fits 2. Diarrhoea 2. Vomiting 3. Fever 4. Loss of appetite

5. Any other specify ___

## Appendix I: Research budget

| ITEM | COST@ITEM | Total cost |
| --- | --- | --- |
| Photocopy of questionnaires | Ksh. 30@x93x4 | 11,160 |
| Electronic weight scales | 2,000 @ set x6 | 12,000 |
| *Moringa oleifera* leaf powder | Ksh.2500 @kg x160 | 400,000 |
| Finger millet flour | Ksh.100@kg x 400 | 40,000 |
| Research assistants (6) | Ksh.10,000 @month x3 months  Ksh.30,000 x6 | 180,000 |
| Training of Research assistants | Ksh.20,000 | 20,000 |
| Travelling allowance | Ksh.5000 per month x3months | 15,000 |
| Nutrient Analysis | Ksh.20,000 x4 | 80,000 |
| Biochemical Analysis | Ksh.30,000 x 2 | 60,000 |
| Data entry | (Ksh.30 per questionnaire x93) x4 | 11,160 |
| Data analysis | Ksh.50,000 x4 | 200,000 |
| Thesis writing (printing and photocopy) | Ksh.20,000 | 20,000 |
| Ethical Review (KUERC) | Ksh.6,000 | 6,000 |
| Ethical Review (NACOSTI) | Ksh.4,500 | 4,500 |
| Miscellaneous | Ksh.100,000 | 100,000 |
|  | **Grand total (Ksh.)** | **1,159,820** |

## Appendix J: Work plan

| **TIME** | **ACTIVITY** |
| --- | --- |
| December 2015-July 2016 | Proposal writing |
| August 2016 | Presentation at Department |
| November 2016 | Submission of proposal to Graduate school |
| July 2017 | Clearance from KUERC |
| August 2017 | Permit from NACOSTI |
| September 2017- April 2018 | Field research |
| May- June 2018 | Data entry and analysis |
| July 2018 | Presentation of results at the department |
| August 2018 | Submission of thesis to Graduate School |
