# Supplementary material for: Effect of Moringa Oleifera fortified porridge consumption on protein and vitamin A status of children with cerebral palsy in Nairobi, Kenya: A randomized controlled trial
Source: PLOS Glob Public Health. 2022 Nov 4;2(11):e0001206. doi: 10.1371/journal.pgph.0001206 (PMC10021702; doi:10.1371/journal.pgph.0001206)
Supplement: Attachment — Submitted filename: RESPONSE TO REVIEWERS.docx [file pgph.0001206.s006.docx]

**RESPONSE TO REVIEWERS**

**Major comments**

**Table 7 is confusing. Are these mean daily nutrient intakes during the 3 months of intervention? Why are they different from the values in Table 2. This difference begs the question of whether the serum retinol and albumin levels seen later are due to the intervention (Moringa) or the improved dietary diversity during the study. Please explain?**

**Did the authors adjust serum retinol for inflammation (elevated C-reactive protein and/or α1-acid glycoprotein) given the high rates of infectious disease in this region? If not, please give reasons. Please see: https://www.ncbi.nlm.nih.gov/pmc/articles/PMC8352745/**

**Assessing vitamin A status using serum retinol is expensive and requires more blood volume draws. The authors should discuss why they did not consider cheaper options— retinol binding protein?**

**Although the tables in the main manuscript are clear, please consider transforming some of the tables with continuous outcomes into figures such as bar plots or boxplots with p-values. Then put these figures in a panel. This will reduce the number of tables.**

**S2 Table (sociodemographic Table) should be included in the main manuscript as Table 1.**

**Current Figure 3 should be placed in the supplementary document.**

**Minor comments**

**Please change “End line” to “endline”**

**Page 11, line 186: change “week day” to “weekday”**

**Page 12, line 209: Change “while” to “but”**

**Page 12, line 223: Change “It” to lower case “it.”**

**There is a typo in this sentence “Data were tested for normality: parametric tests including test” please correct this.**

**Please change to change “Mc Nemar’s” to “McNemar’s Test” throughout**

**Please do not capitalize M in Morbidity in the caption of Table 5**

**Page 16, line 298: Please do not capitalize “Cerebral Palsy”**

**SD (SE) of baseline and endline serum albumin in table 9 disagree with that of table 2 and 8, respectively. Please clarify.**

**Please add a comma instead of a period in this sentence “The rates of undernutrition among the intervention and control group children were 10.52% and 34% respectively while 71.9% and 57.1% of children from intervention and control groups respectively. Were in the normal weight range”**

**Table 11 change “BMI category” to “BMI Z scores”**

**When describing the difference between the interventions and control group at the end of the trial, please explicitly state that these observations are post-study (endline). This information should be in the table title.**

**As clearly demonstrated in the study design in Figure 2, explicitly add the time points of 24-h recall assessment into the “Dietary intake” section.**
